# Porous Metal Current Collectors for Alkali Metal Batteries

**DOI:** 10.1002/advs.202205695

**Published:** 2022-11-27

**Authors:** Jianyu Chen, Yizhou Wang, Sijia Li, Huanran Chen, Xin Qiao, Jin Zhao, Yanwen Ma, Husam N. Alshareef

**Affiliations:** ^1^ State Key Laboratory of Organic Electronics and Information Displays (KLOEID) and Jiangsu Key Laboratory for Biosensors Institute of Advanced Materials (IAM) Nanjing University of Posts and Telecommunications 9 Wenyuan Road Nanjing 210023 China; ^2^ Materials Science and Engineering King Abdullah University of Science and Technology (KAUST) Thuwal 23955‐6900 Saudi Arabia; ^3^ Suzhou Vocational Institute of Industrial Technology 1 Zhineng Avenue Suzhou International Education Park Suzhou 215104 China

**Keywords:** alkali metal anode, current collector, fabrication method, pore structure, porous metal

## Abstract

Alkali metals (i.e., Li, Na, and K) are promising anode materials for next‐generation high‐energy‐density batteries due to their superior theoretical specific capacities and low electrochemical potentials. However, the uneven current and ion distribution on the anode surface probably induces undesirable dendrite growth, which leads to significant safety hazards and severely hinders the commercialization of alkali metal anodes. A smart and versatile strategy that can accommodate alkali metals into porous metal current collectors (PMCCs) has been well established to resolve the issues as well as to promote the practical applications of alkali metal anodes. Moreover, the proposal of PMCCs can meet the requirement of the dendrite‐free battery fabrication industry, while the electrode material loading exactly needs the metal current collector component as well. Here, a systematic survey on advanced PMCCs for Li, Na, and K alkali metal anodes is presented, including their development timeline, categories, fabrication methods, and working mechanism. On this basis, some significant methodology advances to control pore structure, surface area, surface wettability, and mechanical properties are systematically summarized. Further, the existing issues and the development prospects of PMCCs to improve anode performance in alkali metal batteries are discussed.

## Graphical abstract

This review provides significant advances on porous metal current collectors (PMCCs) for alkali metal batteries, including their working mechanism, development timeline, categories, fabrication methods, together with some significant methodology advances on pore structure design. It furthermore presents perspectives for the challenges and opportunities for the fabrication and application of PMCCs.

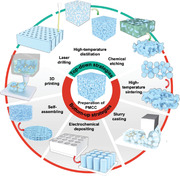

## 1 Introduction

Rechargeable battery as a predominant lightweight power source is essential to the development of human society. Lithium (Li)‐ion batteries occupy 63% of the global battery market by 2020, and the need for portable electronics and electric vehicles is becoming increasingly urgent.^[^ ^1^, ^2^, ^3^, ^4^, ^5^ ^]^ However, the current Li‐ion batteries are still challenging to meet the continuously increased energy density requirements and the rapid development of large‐scale energy storage techniques.^[^ ^6^, ^7^, ^8^ ^]^ In detail, the energy density of a Li‐ion battery is mainly determined by its anode and cathode materials, but the commercially used graphite anodes and transition metal oxide cathodes are almost close to the theoretical capacity ceilings, making it hard to gain more breakthroughs in energy density.^[^ ^9^, ^10^, ^11^ ^]^ Hence, it is of vital importance to develop new battery systems with higher energy densities to compensate or substitute the current Li‐ion batteries.^[^ ^12^, ^13^ ^]^ In the past 20 years, the emerging cathode alternatives, such as sulfur (S), O_2_, and CO_2_, have led to the flourishing development of high‐energy battery systems, i.e., Li–S, Li–O_2_, and Li–CO_2_ batteries.^[^ ^14^, ^15^, ^16^, ^17^, ^18^ ^]^ For anodes, replacing traditional graphite with Li metal or even the high‐reserve and low‐cost Na/K metals, i.e., developing alkali metal batteries, has attracted enormous research efforts due to the high theoretical specific capacities (Li: 3860 mAh g^−1^; Na: 1166 mAh g^−1^; K: 685 mAh g^−1^) and low redox potentials (Li: −3.04 V, Na: −2.71 V, K: −2.93 V vs normal hydrogen electrode) of alkali metals.^[^ ^19^, ^20^, ^21^, ^22^ ^]^

**Figure 1:**
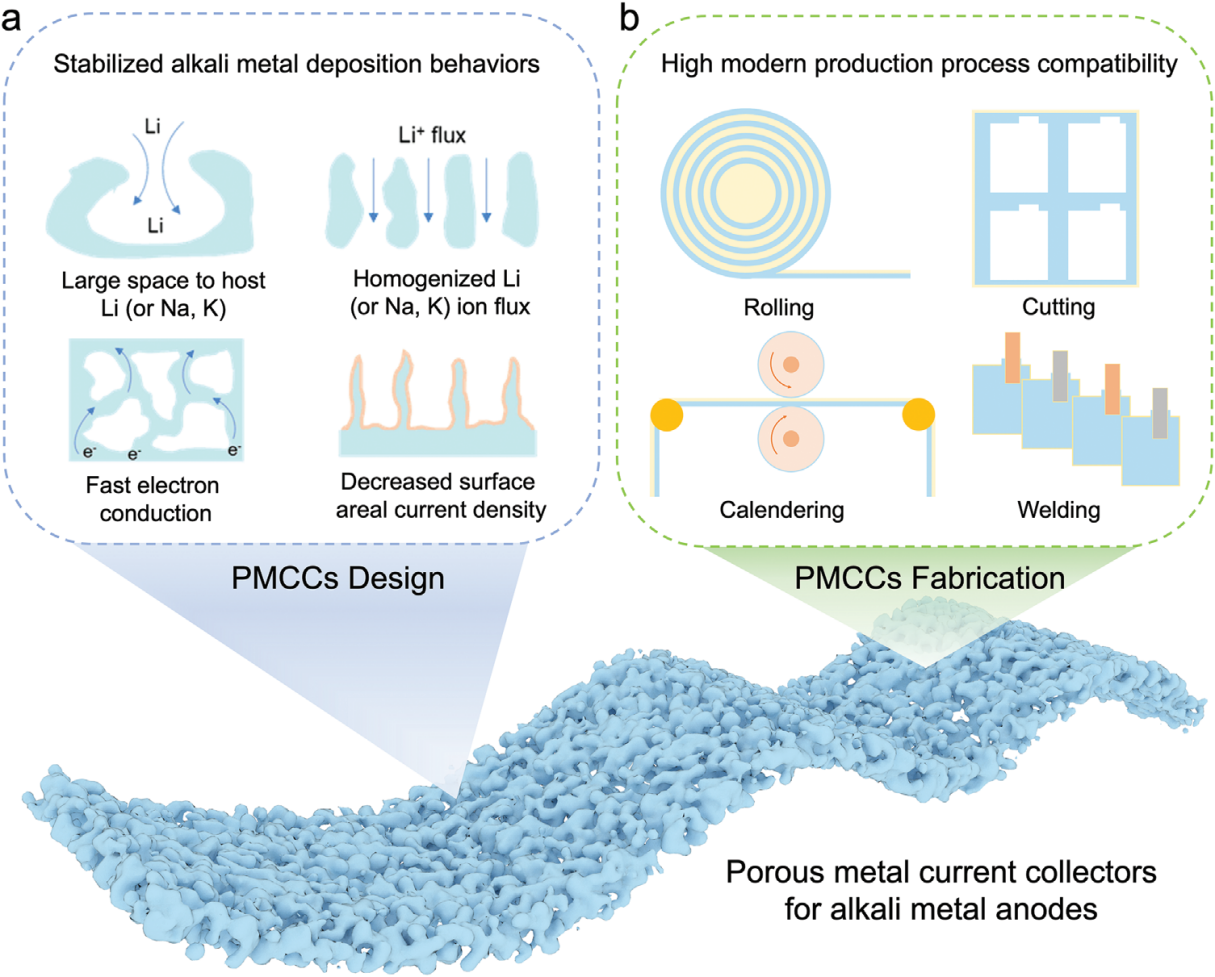
Porous metal current collectors’ (PMCCs) a) design strategies and b) fabrication strategies.

Although the theoretical specific capacity and electrochemical potential of alkali metal are beneficial for achieving high energy density, these alkali metal anodes still face the problem of uneven alkali metal deposition which probably results in dendrite growth, low Coulombic efficiency, and thereby the short lifetime of batteries.^[^ ^23^, ^24^, ^25^, ^26^, ^27^ ^]^ Recently, considerable efforts have been devoted to preventing the growth of dendrites and prolonging the stability of alkali metal anodes, including the strategies of interface regulation,^[^ ^28^, ^29^ ^]^ anode structure engineering,^[^ ^30^ ^]^ electrolyte optimization,^[^ ^30^, ^31^, ^32^ ^]^ stress release,^[^ ^33^ ^]^ and self‐healing mechanism utilization.^[^ ^34^ ^]^ It is generally accepted that the dendrite growth is caused by the uneven current and ion distribution on the anode surface.^[^ ^7^ ^]^ The current collector plays an important role in affecting the performance of electrodes. The design of current collectors could optimize the current density and ion diffusion on anodes. Thus, it has been widely applied to depress the dendrite growth of alkali metal anodes.^[^ ^14^ ^]^ Carbon‐based nanomaterials have multiple allotropes which could be used as the alkali metal anode host. Among them, graphene and carbon nanotubes with sp^2^‐hybridized carbon exhibit excellent physiochemical properties, especially the low‐density characteristic that shows great promise in elevating the energy density of batteries. However, most carbon‐based nanostructures as current collectors will consume a large number of alkali metal ions to form solid electrolyte interphase (SEI) layers because of the large specific surface area of carbon nanomaterials.^[^ ^8^, ^14^, ^20^ ^]^ From the perspective of practical application, employing metal foils (e.g., Cu foil, Al foil) as current collectors for alkali metal anodes could facilitate the integration in the current industrial battery manufacture. However, due to the inhomogeneous distribution of current density and ion concentration on the surface of the normally used planar current collectors, the alkali metal readily tends to grow toward dendrite during the electrodeposition process, resulting in short‐circuit and severe safety hazards.^[^ ^30^ ^]^ The uneven plating/stripping of alkali metal will also cause dendrite growth and lead to low Coulombic efficiency.^[^ ^35^ ^]^ The structural features of porous metal current collectors (PMCCs) are beneficial for homogenizing the distribution of surface current density and ion flux as well as providing sufficient inner space to accommodate the deposited alkali metal, thus effectively alleviating the metal dendrite formation. In addition, due to their metallic nature, developing PMCCs can well fit the modern battery industrial production processes, including rolling, calendering, cutting, and welding (**Figure** **1**). Therefore, fabricating PMCCs is of great potential for promoting the development of high‐energy‐density alkali metal batteries.

This review summarizes the working mechanism, the issues faced by alkali metal batteries, and the guiding principles for designing ideal alkali metal anodes. Then, a systematic survey on PMCCs is provided to highlight the positive role of porous architecture for alkali metal current collectors and to offer a detailed and comprehensive understanding of the structure–performance of PMCCs. First, the fabrication approaches of PMCCs with each of their characteristics, including top‐down strategies (i.e., laser drilling,^[^ ^36^, ^37^, ^38^ ^]^ high‐temperature distillation,^[^ ^39^ ^]^ and chemical etching^[^ ^40^, ^41^ ^]^) and bottom‐up strategies (i.e., high‐temperature sintering,^[^ ^42^, ^43^, ^44^, ^45^, ^46^, ^47^ ^]^ slurry casting,^[^ ^48^, ^49^, ^50^ ^]^ electrochemical deposition,^[^ ^46^, ^51^, ^52^, ^53^, ^54^, ^55^, ^56^, ^57^, ^58^, ^59^, ^60^ ^]^ self‐assembly,^[^ ^61^ ^]^ and 3D printing^[^ ^62^, ^63^ ^]^) have been reviewed and discussed. Subsequently, the advances of PMCCs according to their composite structures in macroscale size, i.e., nonfreestanding structure^[^ ^7^, ^48^, ^49^, ^50^ ^]^ and freestanding structure;^[^ ^42^, ^43^, ^44^, ^45^, ^46^, ^47^, ^61^, ^62^, ^63^ ^]^ specific designs in microscale size, i.e., disordered,^[^ ^40^, ^41^, ^42^, ^43^, ^44^, ^51^, ^52^, ^53^, ^54^, ^64^, ^65^, ^66^, ^67^, ^68^ ^]^ ordered,^[^ ^36^, ^37^, ^38^, ^56^, ^57^, ^61^, ^62^, ^63^, ^68^, ^69^ ^]^ elastic,^[^ ^48^, ^70^, ^71^ ^]^ gradient pore design,^[^ ^47^ ^]^ bidirectional,^[^ ^45^ ^]^ and functional design, i.e., lithiophilic design,^[^ ^57^, ^58^, ^59^, ^72^, ^73^, ^74^, ^75^, ^76^, ^77^, ^78^, ^79^, ^80^, ^81^, ^82^ ^]^ and gradient‐designed PMCCs are summarized in detail with the discussions of the corresponding structure–performance relations.^[^ ^83^, ^84^, ^85^, ^86^, ^87^ ^]^ Finally, the remaining challenges and future perspectives of PMCCs have been concluded and presented based on the reported research as well as our own understanding and research experiences. We deeply hope that this review will provide essential knowledge to guide the structure design of PMCCs and thereby promote the development of alkali metal batteries.

## 2 Working Principles of Alkali Metal Anodes

The batteries with alkali metal anodes are considered as ideal candidates for future energy storage because of their low density, high theoretical specific capacity, and low potential. Over the past 40 years, researchers have been trying to apply Li, Na, and K metals in rechargeable batteries, such as matching them with intercalation cathode materials to achieve a high voltage window or high‐specific‐capacity materials, like S, O_2_, and CO_2_, to elevate the battery energy density.^[^ ^27^, ^88^ ^]^ The theoretical energy densities of Li–S and Li–O_2_ batteries are as high as 2600 and ≈3600 Wh kg^−1^, respectively, making Li‐metal‐based batteries highly promising next‐generation high‐energy‐density batteries and receiving extensive attention. The Li–CO_2_ battery has a high theoretical energy density of ≈1876 Wh kg^−1^ and theoretical equilibrium potential.^[^ ^89^ ^]^ In addition, advanced Li–CO_2_ battery technology can effectively improve the utilization of CO_2_ and indirectly reduce CO_2_ emissions.

Similar to the alkali‐ion battery, the alkali metal battery also relies on the migration of cations between the cathode and anode to realize reversible energy storage and release. The main difference between the alkali‐ion battery and the alkali metal battery lies in the anode materials and the working mechanism.^[^ ^90^ ^]^ In the alkali‐ion battery system, cations react with the anode through insertion, alloying, or conversion reactions and finally form compounds during the charging process. The alkali metal ions are then released into the electrolyte during the discharging process. Taking a commercial Li‐ion battery as an example, the Li ions are reversibly inserted into and extracted from the graphite anode during the charging and discharging processes, respectively. Based on the different working mechanisms, the anode materials for alkali‐ion batteries can be divided into intercalation, alloying, and conversion types. For alkali metal batteries, alkali metals are used as anodes, and they switch between the states of alkali ions and alkali metal during the charging and discharging processes, respectively. Alkali metal atoms tend to aggregate on planar metal surfaces or planar current collectors during the nucleation, and some metal atoms might grow into dendrites, which may result in short circuits and even the explosion of the battery. Moreover, alkali metal dendrites tend to separate from their roots, which causes the top metal to be isolated as “dead metal” and thus lose electrical contact. Therefore, strategies covering the host, electrolyte, and/or separator are strongly required to construct a highly stable and recyclable anode for the practical application of alkali metal batteries.

In fact, commercially available cathode and anode both require a current collector to load the active materials in different battery systems. Exemplified with intercalation‐type anode materials, the commercial graphite electrode, the commonly used one, is composed of an ≈8 µm thick Cu foil with 100 µm thick graphite layers coated on both sides.^[^ ^91^ ^]^ It can pair with different kinds of cathodes to achieve high energy densities. And when introducing the same thickness of Li foils on the thin Cu foil, it can theoretically match more cathode materials and achieve higher energy density due to the large specific capacity and low density of Li metal. However, unstable Li metal will lead to poor electrochemical performance due to the dendrite growth and significant volume changes of Li. Therefore, the skillful design of metal host with porous structure is essential to improve the stability of the alkali metal anode and meantime guarantee the high specific capacity.

## 3 Basics of PMCCs

### 3.1 Working Principles of PMCCs

In a rechargeable alkali‐metal‐anode system, the uneven electric field and ion flux on planar conductive substrates will result in uncontrolled alkali metal deposition and even dendrite growth.^[^ ^88^, ^89^, ^90^, ^91^, ^92^, ^93^, ^94^, ^95^ ^]^ Therefore, rationally designing the current collector structure can help regulate the nucleation of alkali metal and control the morphology of deposited alkali metal. One of the most effective approaches is constructing a porous conductive substrate to dissipate the local current density and uneven ion flux, finally contributing to a homogeneous formation of alkali metal and obtaining a dendrite‐free alkali metal anode.

#### 3.1.1 Homogeneous Current Density Distribution

The generally acceptable “space charge theory” used to explain the relationship between the dendrite formation and the current density distribution was proposed by Chazalviel.^[^ ^16^, ^95^ ^]^ Specifically, the model assumed that the metal ion is reduced in a dilute electrolyte solution, and the cation gradient exists between the surface of the anode and cathode. During the rapid discharging process, the ionic concentration drops near the surface of the anode, generating an electric field and local space‐charge region on the interface between the electrolyte and surface of the anode. As shown in Equations (1)–(3), the local ionic concentrations near the electrode surface drops to zero at a short time (*τ*_s_) when the applied current density reaches the critical value J*. At this time, the dendrites start to form. The *J* is the applied current density; *µ*_a_ and *µ*_c_ refer to the mobilities of anions and cations, respectively. The *µ*, *e*, and *D* are assumed to be independent of concentration, electronic charge, and diffusion constant. *C*_0_ refers to the initial concentration of cation, and *L* is the distance between the electrodes

(1) $$\frac{\partial C}{\partial x}\left(x\right)=\frac{J\mu_{a}}{eD\left(\mu_{a}+\mu_{c^{+}}\right)}$$

(2) $$J^{*}=2eC_{0}D\left(\mu_{a}+\mu_{c^{+}}\right)/\mu_{a}L$$

(3) $$\tau_{s}=\pi D{\left\{\frac{eC_{0}\left(\mu_{a}+\mu_{c^{+}}\right)}{2J\mu_{a}}\right\}}^{2}$$

**Figure 2:**
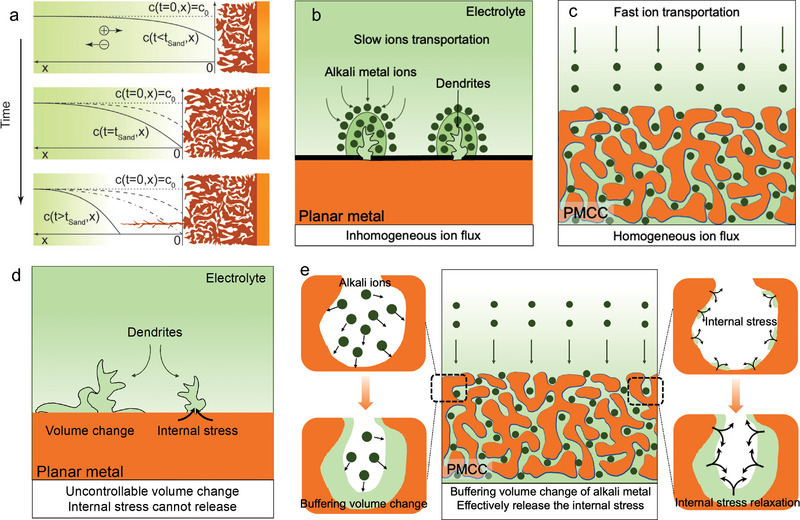
Working principles of PMCCs. a) The classical theoretical model for time‐dependent alkali metal deposition during different concentration polarizations. Reproduced with permission.^[^ ^95^ ^]^ Copyright 2016, The Royal Society of Chemistry. b,c) Schematic diagram of principle for ion transportation behavior by planar metal (b) and PMCC substrate (c). d,e) Schematic diagram of volume change and stress‐driven dendrite growth behavior in planar metal (d) and PMCC substrate (e).

According to the description of Sand's time (**Figure** **2**a), the ion concentration gradient will elevate on the surface of the planar electrode, leading to the low ion concentration on the anode surface and accelerating the dendrite growth of alkali metal. Therefore, transforming the planar electrode into a 3D porous electrode can effectively increase the specific surface area of the electrode and reduce the distribution of local current density to prolong the initial growth time of dendrites, namely increasing the *τ*_s_.

#### 3.1.2 Homogeneous Alkali‐Ion Diffusion

The transportation of alkali ions from the electrolyte to the anode surface is essential during the discharge process and plays a vital role in electrochemical deposition.^[^ ^88^ ^]^ The general verdict is that uneven alkali‐metal‐ion flux and sluggish ion transport rate on the surface of current collectors usually induce inhomogeneous alkali metal nucleation and growth, which may further generate the undesired dendrite and thereby the battery failure.^[^ ^93^, ^94^ ^]^ Hence, to regulate the homogeneous transport of alkali metal ions to accomplish highly stable alkali metal anodes, the ideal principle strategies are allocated to regulate homogeneous alkali‐ion flux and expedite the alkali‐ion diffusion rate. In addition, the current collectors’ structure significantly influences the alkali‐ion flux, finally determining the nucleation and subsequent growth of alkali metal. A conventional planar current collector with randomly dispersed protuberances on its surface easily causes charge accumulation in local areas, resulting in high‐current‐density distribution (Figure 2b). Additionally, according to the “space charge theory,” the concentration of the cation at the interface of electrolyte/anode will drop to zero during the applied high current density. Decreasing the current density could effectively increase the Sand's time. Meanwhile, the inhomogeneous electric field distribution will result in an uneven alkali‐ion flux at the beginning of the plating process.^[^ ^95^ ^]^ However, the porous conductive structure with well‐distributed pores not only reduces the effective current density but also regulates the homogeneous alkali‐metal‐ion flux (Figure 2c). Moreover, 3D porous metal current collectors have a high surface area, which can change alkali metal ions’ diffusion path from 2D to 3D. The high porosity can significantly facilitate electrolyte penetration in the skeleton, narrow the alkali‐ion concentration boundary layer, and reduce the alkali metal ions’ concentration polarization.^[^ ^92^ ^]^

#### 3.1.3 Buffering Volume Change of Alkali Metal

Compared with alkali metal batteries, mobile‐ion batteries have anode hosts that can accommodate the volume change during battery operation. By contrast, the hostless nature of alkali metal anodes results in infinite volume expansion during the deposition process of alkali metals.^[^ ^88^, ^90^ ^]^ As discussed above, the inhomogeneous distribution of charge and ion concentration on the surface of a planar alkali metal or a planar current collector can trigger inhomogeneous metal deposition. Alkali metals tend to be deposited in rod‐like, tree‐like, and moss‐like morphologies and lead to very large volume expansion (Figure 2d).^[^ ^94^ ^]^ Actually, the practical volume change of alkali metals at the same areal loading is determined by several factors such as density, molar volume, and charging/discharging current density. Compared with metallic Li, Na and K metals have a larger molar volume, which leads to a larger volume change during cycling. Therefore, the potential cyclability and stability problems in Na‐ and K‐metal‐anode systems will be more severe. The ample pore space of PMCCs can effectively buffer the “swelling and shrinking” of alkali metals during the repeated plating and dissolution process (Figure 2e).

#### 3.1.4 Stress Relaxation

Stress plays a vital role in influencing the deposition behavior of alkali metals. Internal stresses are generated during metal plating, which in turn affect the deposition behavior of alkali metals.^[^ ^70^ ^]^ The migration process of alkali metal atoms through grain boundaries under nonequilibrium growth conditions is the decisive factor for internal stress accumulation. The stress on the planar electrode cannot be effectively released, which will drive the deposited metal growth into metal whiskers, metal mosses, or metal dendrites (Figure 2d).^[^ ^96^ ^]^ Constructing a porous structure can effectively alleviate the internal stress induced by metal deposition.^[^ ^70^ ^]^ Specifically, the abundant pores of PMCCs can not only accommodate the large volume changes caused by alkali metal deposition/dissolution, but also release the stress generated from the deposited alkali metals inside the pore structure (Figure 2e). For example, a 3D soft metal host and elastic host can accommodate the volume change and effectively release the stress, finally mitigating the dendrite growth of Li metal.^[^ ^62^, ^70^ ^]^

### 3.2 History of PMCCs

**Figure 3:**
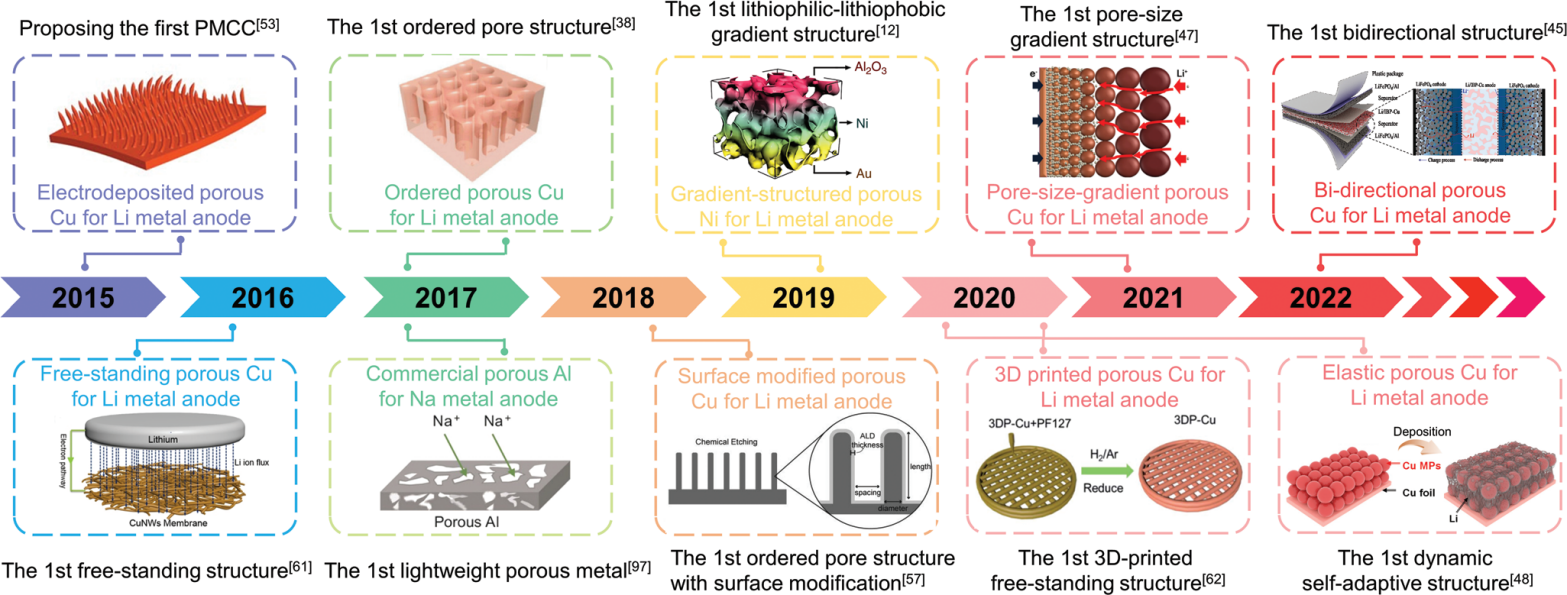
Timeline of the development of PMCCs for alkali metal anodes. Reproduced under the terms of the CC‐BY Creative Commons Attribution 4.0 International license (https://creativecommons.org/licenses/by/4.0).^[^ ^53^ ^]^ Copyright 2015, The Authors, Published by Springer Nature. Reproduced with permission.^[^ ^61^ ^]^ Copyright 2016, American Chemical Society. Reproduced with permission.^[^ ^38^ ^]^ Copyright 2017, Wiley‐VCH. Reproduced with permission.^[^ ^97^ ^]^ Copyright 2017, American Chemical Society. Reproduced with permission.^[^ ^57^ ^]^ Copyright 2018, Wiley‐VCH. Reproduced under the terms of the CC‐BY Creative Commons Attribution 4.0 International license (https://creativecommons.org/licenses/by/4.0).^[^ ^12^ ^]^ Copyright 2019, The Authors, Published by Springer Nature. Reproduced with permission.^[^ ^62^ ^]^ Copyright 2020, The Royal Society of Chemistry. Reproduced with permission.^[^ ^47^ ^]^ Copyright 2021, American Chemical Society. Reproduced with permission.^[^ ^48^ ^]^ Copyright 2020, American Chemical Society. Reproduced with permission.^[^ ^45^ ^]^ Copyright 2022, Wiley‐VCH.

Over the past decade, researchers have explored viable PMCCs to address the abovementioned problems of alkali metal anodes. **Figure** **3** shows the timeline for the development of PMCCs with their associated material systems, pore structures, fabrication methods, and additional functions from 2015 to 2022. The pioneering study of employing PMCC for stabilizing alkali metal anode was reported by Yang et al. in 2015. They applied electrodeposited porous Cu fiber on planar Cu foils to construct a composite porous metal current collector for stabilizing Li‐metal anodes, illustrating the relationship between the electroactive surface area induced by porous structure and the electrochemical plating behavior of Li metal.^[^ ^53^ ^]^

After that, PMCCs have witnessed rapid development in recent several years. Considerable efforts have focused on the construction and design of PMCCs with a composite structure. Distinguishing from the above composite structures, Yu and co‐workers first proposed a new method to construct a freestanding porous metal current collector for stabilizing Li‐metal anodes in 2016. During this period, PMCCs essentially maintain a disordered pore structure whether in a composite or freestanding system.^[^ ^61^ ^]^

In 2017, Wang et al. applied laser drilling to generate ordered pore structures on Cu foils for the first time. They investigated the relationship between pore size/depth/spacing and electrochemical performances.^[^ ^38^ ^]^ In the same year, Liu et al. extended the research of PMCCs to Na‐metal anode based on commercial porous Al foils. This is a meaningful material in the field of Na‐metal anode which is also a milestone in the history of alkali metal batteries. The discovery of a porous Al current collector enriches the types of PMCC materials, dramatically reduces the mass of inactive substances, and effectively improves the energy density of Na metal batteries.^[^ ^97^ ^]^

After that, the development of porous metal carriers is not limited to the design and modification of pore structures. Great efforts have been made to coordinate surface lithiophilic modifications, lithiophilicity–lithiophobicity gradient designs, and pore‐size gradient designs of PMCCs. For example, Dasgupta and co‐workers employed the atomic layer deposition (ALD) technique to deposit ZnO onto vertically aligned Cu pillars with ordered pore structures to obtain a lithophilic‐modified porous metal current collector.^[^ ^57^ ^]^ Pu et al. developed a well‐designed Al_2_O_3_–Ni–Au gradient‐distributed porous metal current collector in 2019, which effectively improved the uniformity of Li metal deposition inside the PMCCs.^[^ ^12^ ^]^ In 2021, Lee et al. designed a pore‐size gradient porous metal current collector to study the relationship of pore size with Li metal deposition behavior.^[^ ^47^ ^]^

Besides, based on the perspective of dendrite growth theory, researchers have revealed the effects of various factors on dendrite nucleation and growth from different aspects, such as charge distribution theory, distribution of alkali‐metal‐ion flux, and stress generation/release. Ma et al. reported a polymer–Cu powder hybrid porous metal current collector and discussed the mechanism of releasing residual stress by PMCCs with elastic function, which exhibited the elastic ability to accommodate the deposition of Li metal.^[^ ^48^ ^]^ In 2021, Dinga et al. developed a 3D printing method to directly fabricate the PMCCs for the first time. With the help of 3D printing technology, the pore structure and volume of PMCCs can be precisely controlled by computer simulation.^[^ ^62^ ^]^

After years of development, advanced structural design, advanced preparation methods, and the introduction of excellent additional functions are all accelerating the progress of high‐performance research on alkali metal anodes. However, for practical application, the process of bringing alkali metal batteries from the button cell to a multilayer stacking pouch cell is still full of tremendous challenges. Compared with the laboratory‐level button battery with a single electrode design, commercial batteries need to improve the battery's energy density by parallel electrodes. More recently, Ma and co‐workers presented the bidirectional PMCCs with both sides accommodating Li plating/stripping for the first time. Using this new design, the bidirectional PMCCs could realize a high energy density elevation, exhibiting great promising practical applications for next‐generation high‐energy‐density Li metal batteries.^[^ ^45^ ^]^

### 3.3 Fabrication Strategies of PMCCs

Alkali metals with high reactivity and thermodynamic instability will react with electrolytes to form an unstable SEI layer.^[^ ^98^ ^]^ Hence, electrochemical stability must be considered when metal materials are selected as current collectors. Metal foils are the most widely used current collectors in lithium‐ion batteries because of their excellent conductivity and mechanical properties.^[^ ^96^, ^99^ ^]^ In the cathode, the corrosive resistance of metal under a high voltage of 2–5 V versus M/M^+^ (M refers to the alkali metal) should be considered for the current collector. Thus, Al and Ti are good choices due to the inert layer formed on their surfaces under high potential.^[^ ^99^, ^100^ ^]^ The challenge is the alloying reaction with alkali at a low potential below 2 V versus M/M^+^ when it comes to the anode side. Thus, Cu foil with excellent electrochemical stability under low potential is widely used as the anode current collector for alkali metal batteries. As discussed above, planar metal electrodes with a low surface area will result in uneven current density and ion flux distribution, finally leading to poor electrochemical performance. Hence, it is essential to construct a porous metal current collector for stabilizing alkali metal anodes with better cycling and safety performance.

**Figure 4:**
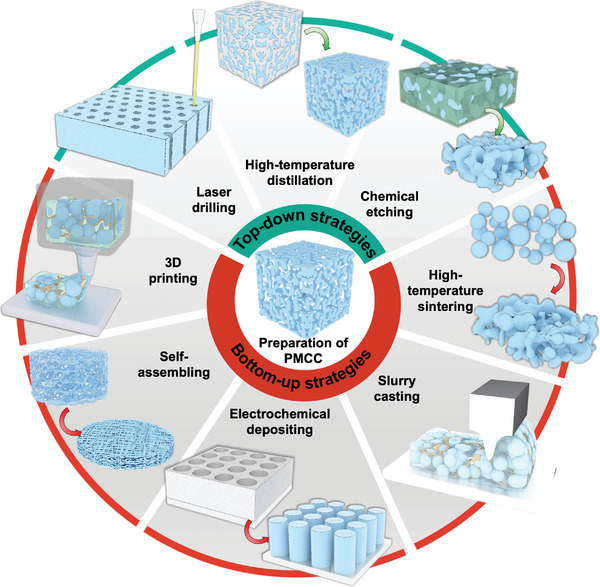
Overview of strategies used to fabricate PMCCs.

The strategies of fabricating PMCCs for alkali metal anodes can be divided into top‐down strategies (i.e., laser drilling, high‐temperature distillation, and chemical etching) and bottom‐up strategies (i.e., high‐temperature sintering, slurry casting, electrochemical deposition, inverse opal templating, and 3D printing), as shown in **Figure** **4**.

#### 3.3.1 Top‐Down Strategies

Top‐down techniques are widely used strategies to develop PMCCs for alkali metal anodes. Generally, metal or metal alloy films are acquired as raw materials for the following top‐down pore structure fabricating. Laser drilling, i.e., irradiation with laser pulses, transforms a suitable metal substrate into porous metal current collectors. Laser techniques allow the fabrication of porous metal with regular pore structure directly, meanwhile performing in the form of hybrid electrodes combining polymer templates or customizable patterns. Guo and co‐workers reported the typical laser‐induced PMCCs with regular and adjustable pore architecture.^[^ ^38^ ^]^ In their study, vertically aligned microchannels with different pore diameters and pore depths can be obtained by a laser microprocessing system. As a result, regulating the geometry of the Cu electrode can effectively reduce the current density distribution of the electrode surface, induce homogenous Li deposition, and improve the electrochemical properties of the battery. For laser drilling, the fine regulation of the pore structure of PMCCs relies heavily on the precision and power of the laser drilling machine.

High‐temperature vacuum distillation is a method to obtain PMCCs, which utilizes the difference of boiling point between different metal elements to remove the impurity elements. Qian and co‐workers demonstrated an environment‐friendly high‐temperature vacuum distillation approach to fabricate the PMCCs from Cu–Zn alloy.^[^ ^39^ ^]^ They adjusted the distillation temperature and time to regulate the pore structure of PMCCs. The as‐prepared PMCCs not only inhibit the growth of Li dendrite but also improve the electrochemical performance of Li metal batteries.

The chemical/electrochemical etching method can effectively acquire porous metal current collectors, similar to the high‐temperature distillation method. Notably, Yang and co‐workers developed a series of chemical/electrochemical dealloying methods to prepare porous Cu current collectors.^[^ ^40^, ^41^ ^]^ By etching brass tape in an acidic solution or electrochemical conditions, the compact structure with uniform and abundant pores enhances the wettability of the electrolyte, decreases the current density of the surface, and induces the formation of a homogenous SEI layer. As a result, the porous Cu current collectors exhibit excellent cycling and rate performances.^[^ ^73^, ^74^ ^]^

PMCCs fabricated by the top‐down method is directly derived from bulk metal, which ensures excellent conductivity and structural stability and is thus beneficial for elevating the electrode performance. However, the top‐down approach usually cannot avoid the huge waste of raw materials and lacks the elemental composition regulation for PMCCs.

#### 3.3.2 Bottom‐Up Strategies

High‐temperature sintering is a facile and low‐cost method for fabricating flexible PMCCs on a large scale. He and co‐workers selected NaCl as a removable template and sintered it with Cu powder to obtain a porous Cu current collector. The removable NaCl increases the porosity of porous Cu, accommodating a large amount of Li to be filled in the pore structure. Besides, Cu carbonate particles based on mixing a solution of Cu(NO_3_)_2_ with Na_2_CO_3_ are chosen as the building block of porous Cu electrodes. After calcination and reducing the Cu carbonate particles, the porous metal host is easily formed with high surface area and conductivity to achieve a better Li‐metal anode performance.^[^ ^46^ ^]^ High‐temperature sintering technology is beneficial to improving the conductivity and integration of PMCCs, which plays a significant role in enhancing the rate performance of Li metal composite anodes. However, high‐temperature sintering inevitably requires the use of vacuum, protective atmospheres, and reductive gases, which not only creates safety hazards but also indirectly increases the material manufacturing costs.

To our knowledge, the slurry‐casting method is low cost and time effective and is widely used in the scalable fabrication of electrodes in batteries. The usage of polymer binders, i.e., poly(vinylidene fluoride), styrene‐butadiene rubber, carboxymethyl cellulose, and poly(acrylic acid), is usually selected for the slurry‐casting process.^[^ ^101^ ^]^ Thus far, researchers have started to explore PMCCs by mixing conductive metal particles with binders. Ma and co‐workers developed a slurry‐casting method to construct a dynamic porous Cu current collector. The dynamic porous Cu electrodes effectively alleviate the stress by changing the stacking density of Cu particles during Li plating/stripping, suppressing the growth of Li dendrite, present excellent stability and cycling performance during long‐term Li plating/stripping. Besides, introducing lithiophilic coating through the slurry‐casting process could decrease the nucleation overpotential, which is beneficial for uniform Li plating and stripping.^[^ ^48^ ^]^ The slurry‐casting method is a well‐established commercial electrode preparation technology, which has significant advantages in preparing large‐scale electrodes. Nevertheless, for some specific electrode preparation processes, such as the preparation of stabilized Li metal powder and Cu powder composite electrodes, the use of binders and organic solvents is inevitable, which will not only cause contamination but also add the additional mass of inactive materials.

The electrochemical deposition technique plays an essential role in the construction of PMCCs, which shows various advantages of being simple, inexpensive, reproducible, and damage‐free. Metal ions tend to grow in the form of dendrites without restriction in the process of electrochemical reduction. With the help of a removable template, the reduced metal can realize steady growth on the conductive substrate. The earliest report consisting of the electrochemically assisted template growth of the preparation of PMCCs dates back to 2006. Tarascon and co‐workers developed a novel electrode design through assisted template growth of Cu nanorods onto a Cu substrate. Subsequently, the active material deposited in the porous Cu current collector obtained ultrahigh electrochemical performance improvement.^[^ ^102^ ^]^ Following this electrode design, Dasgupta and co‐workers demonstrated vertically aligned Cu pillar arrays with lithiophilic ZnO coatings as hosts for Li metal deposition and dissolution. This highly ordered Cu pillar avoids the closed pore structure in the presence of tortuous disordered geometries, which facilitate accommodation of more Li deposition. Leveraging the synergistic effects of interface modification and the highly ordered geometry, the PMCC‐based Li‐metal anodes achieved a high electrochemical performance.^[^ ^57^ ^]^ In addition, the strategy of applying opal stacking structure as a template has been used to achieve porous metal current collectors by using electrochemical deposition. For instance, Deng and co‐workers developed an inverse opal template method to construct an ordered macroporous Cu current collector. Specifically, by dispersing the methyl methacrylate (MMA) monomer into 2,2‐azobisisobutylamidine dihydrochloride solution under a heating condition, the poly(methyl methacrylate) (PMMA) microspheres with a diameter of about 450 nm were produced. The PMMA colloidal template was prepared by an electrophoresis method for subsequent Cu deposition. After dissolving the PMMA, the 3D‐ordered porous Cu current collector was obtained. As a result, the ordered PMCC with the pore size of 450 nm can achieve high Coulombic efficiency of 93.3% for 750 h at a current density of 0.2 mA cm^−2^ with a low electrochemical polarization (<30 mV).^[^ ^60^ ^]^ Combining electrochemical deposition techniques with removable templates can precisely control the pore structure of PMCC, but this method also inevitably leads to waste of templates. Therefore, the use of more advanced electrochemical deposition techniques without removable rigid templates is considered as one of the ideal strategies to obtain PMCCs. For example, Hou and co‐workers developed a 3D porous Cu through a hydrogen‐bubble‐dynamic‐template‐based electrochemical deposition method. The pore structure and volume could be adjusted by manipulating the concentration of the plating solution, deposition time, and surfactant additive. Benefiting from these merits, the 3D porous Cu structure revealed the ability to decrease the local current density and could accommodate the volume change of Li metal during the repeated deposition and dissolution process. As a result, the optimized porous Cu current collectors achieve high electrochemical reversibility and stability. Highly stable Coulombic efficiency is achieved in half‐cell at different rates with a cycling capacity of 1 mAh cm^−2^. The Li–3D Cu composite anode exhibited stable cycling for 145 h at a current density of 3 mA cm^−2^.^[^ ^63^ ^]^ It is well‐known that the electrochemical deposition technique is considered as one of the most widespread methods to construct specific porous metal structures due to its precise tuning of electrochemical parameters. However, in view of the large‐scale production, the wastewater treatment should be a big problem for the electroplating industry.

The self‐assembly method is a widely adopted technique in the field of micro‐ and nanomaterials to construct macroscopic porous electrodes.^[^ ^61^ ^]^ Yu and co‐workers developed a series of freestanding metal nanowires as current collectors to accommodate Li metal using the self‐assembly method. Specifically, the Cu nanowire (Cu NW) current collectors realized a high Li metal accommodation of 7.5 mAh cm^−2^, and displayed a high Coulombic efficiency of 98.6% with a small voltage polarization of ≈ 0.04 V after 200 cycles due to the high conductive Cu NW network and suppressed Li dendrite growth. The Cu–Ni nanowires (Cu@Ni NWs) as a highly lithiophilic host also presented a superior electrochemical performance in a symmetrical and full‐cell system.^[^ ^103^ ^]^ The as‐prepared Li–Cu@Ni composite anode delivers a high gravimetric capacity of ≈1882 mAh g^−1^. The composited Li‐metal anode displays a flat voltage polarization of 140 mV for stably cycling for more than 500 cycles. Self‐assembly techniques generally use metal nanowires as the building blocks to construct porous metal electrodes. Owing to their high conductivity and flexibility, the metal‐nanowire‐based PMCCs exhibit high structural reversibility, rate capability, and cycling life for Li‐metal anodes. However, the self‐assembly method is inconvenient for large‐scale electrode fabrication and is strongly limited by the quality of metal NWs. Considering the requirements of low cost and industrial application of the materials for battery technologies, the self‐assembly method is now still limited in lab research.

Oxidation–reduction is one of the earliest methods used to prepare PMCCs, which generally can be divided into two steps: 1) oxidation of the primary material; 2) reduction of the oxides in PMCCs. Guo et al. reported a porous Cu current collector by first growing Cu(OH)_2_ sub‐micrometer fibers on the surface of Cu foil through water oxidation and then obtaining a porous Cu structure after the reduction of Cu(OH)_2_.^[^ ^53^ ^]^ Owing to the numerous protuberant tips and the reduced current density provided by the porous structure, the porous Cu current collector exhibited homogeneous Li metal deposition which stably runs up to 600 h with a high average Coulombic efficiency of ≈98.5%. Later, numerous studies were published using the oxidation–reduction method, which also achieved exceptional electrochemical stability. However, the high density of the substrate and the inability to precisely regulate the pore structure and volume severely limit the practical application of PMCCs prepared by the “oxidation–reduction” method.

Compared with the traditional fabrication process, 3D printing technology has received extensive attention in recent years. Applying 3D printing technology to the preparation of conductive metal electrodes, arbitrary geometry and structure of electrodes could be precisely customized through a fast prototyping process. Interestingly, 3D printing technology can be adapted to directly print integrated electrodes with high porosity. Preparing metal electrodes by 3D printing is different from other conventional 3D printing technologies. It needs a high‐power laser beam working on the surface of fusible powder materials to sinter them into a solid‐state structure. Recently, Ding and co‐workers reported a 3D‐printed porous Cu current collector by a novel extrusion‐based direct ink writing approach. This newly developed strategy can obtain the porous Cu current collector with ordered microchannels of 100–300 µm. As a result, the porous Cu current collector can accommodate an ultrahigh Li deposition of 20 mAh cm^−2^ and exhibit high electrochemical performance.^[^ ^62^ ^]^

Overall, the PMCCs obtained from the abovementioned top‐down or bottom‐up methods have been proven to effectively suppress the Li dendrite growth by regulating the current density and ion flux during Li metal plating. The PMCCs obtained by all these methods can also be applied to Na‐ and K‐metal anodes. In addition, the material system of PMCCs can be extended to other metals, such as Al due to its nonalloying reaction with Na and K metals at low potential. While these methods show highly stabilized alkali metal hosts with different pore structures, it is still a big challenge to develop more advanced techniques to obtain ideal PMCCs with the advantages of tunable pore structure, low cost, eco‐friendly, and scale‐up preparation. Therefore, developing PMCCs to address the adverse issues of alkali metal anodes should synergistically consider the strategies of electrode design, interfacial protection, and the optimization of electrolytes.

## 4 Advanced PMCCs for Li‐Metal Anodes

The electrochemical stability of PMCCs plays a significant influence in the electrochemical performance of Li‐metal anode. The main challenge is the probably alloying reaction with Li at the low electrochemical potential below 2 V versus Li/Li^+^.^[^ ^99^ ^]^ Therefore, Cu, Ni, and stainless steel are usually selected as anode current collectors due to their high electrochemical stability under low potential. However, directly using these planar metal foil as anode current collectors will cause inhomogeneous Li‐ion flux dispersion and formation of tip effect, finally inducing the Li dendrite growth. Therefore, tailoring planar metal structures into 3D or porous architecture can effectively balance the charge and mass transfer. In this section, we highlight the different PMCCs based on macroscale electrode structure design, pore structure design, and additional function design.

### 4.1 Macroscale Electrode Structure Design

Conventional planar electrode surfaces are normally coarse, which induces unbalanced alkali ions and charge transfer between the electrolyte and anode surface with uneven current distribution and ion flux. Alkali metal ions prefer to accumulate on the disordered protuberances, resulting in a large amount of charge accumulation and then triggering the formation and propagation of alkali metal dendrites. According to “space charge theory,” the current density of the electrode surface is a decisive factor that influences the growth of alkali metal dendrites. Decreasing the local effective current density of the electrode surface can prolong the dendrite growth time. Therefore, designing conventional planar structures into the porous structure is an effective strategy to reduce the local effective current density and thus extend the cycling performance of alkali metal anodes. In this section, we systematically discuss the design of electrode structures with macroscale size for PMCCs including nonfreestanding structures and freestanding structures.

#### 4.1.1 Nonfreestanding Structure

**Figure 5:**
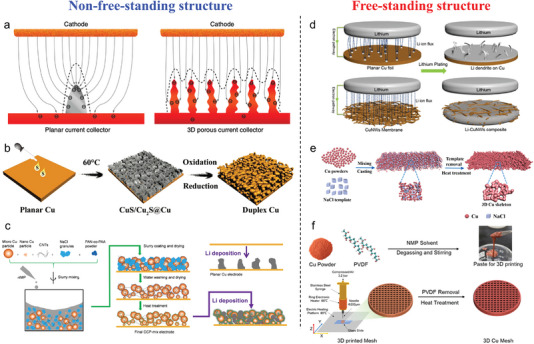
Advanced PMCCs based on macroscale electrode structure design. a) Schematic illustration of Li deposition behaviors on planar current collector and 3D current collector. Reproduced under the terms of the CC‐BY Creative Commons Attribution 4.0 International license (https://creativecommons.org/licenses/by/4.0).^[^ ^53^ ^]^ Copyright 2015, The Authors, Published by Springer Nature. b) Schematic illustration of the procedures to prepare a porous duplex Cu current collector. Reproduced with permission.^[^ ^67^ ^]^ Copyright 2020, Wiley‐VCH. c) Schematic illustration of the fabrication process of the porous lithiophilic and conductive PMCC, Li deposition mechanisms on planar Cu, and porous lithiophilic and conductive PMCC. Reproduced with permission.^[^ ^50^ ^]^ Copyright 2019, The Royal Society of Chemistry. d) Schematic illustration of Li deposition behaviors on planar current collector and porous Cu nanowire current collector. Reproduced with permission.^[^ ^61^ ^]^ Copyright 2016, American Chemical Society. e) Schematic illustration of the procedures to prepare a porous Cu current collector. Reproduced with permission.^[^ ^46^ ^]^ Copyright 2018, American Chemical Society. f) Schematic illustration of the procedures to prepare a 3D printed porous Cu current collector. Reproduced with permission.^[^ ^65^ ^]^ Copyright 2021, American Chemical Society.

According to the description of Sand's time, the *τ*_s_ predicts the threshold time for the initial growth of dendrites, which means that the time of dendrite growth can be prolonged by reducing the effective current density. Therefore, an effective route is to adopt the porous conductive substrate as the host for Li‐metal anodes. As mentioned in Section 3.2, Guo and co‐workers found that the metallic Li is apt to first generate a small protuberance on the planar Cu substrate at the initial nucleation step.^[^ ^53^ ^]^ More nucleation sites are constructed by introducing a non‐self‐supporting structure (i.e., planar electrodes and porous conductive metal layers) of 3D porous Cu. Li metal tends to grow on the porous Cu layers and gradually fills up the pores formed by the porous Cu fibers, as shown in **Figure** **5**a. After that, a series of research works reported the preparation methods to obtain PMCCs by constructing porous metal layers on the surface of planar Cu foils. As reported by Lin et al., a duplex‐type porous metal current collector with an ant‐nest‐like porous Cu layer and a dense Cu substrate acts as the stable host for the Li‐metal anode (Figure 5b).^[^ ^67^ ^]^ Specifically, a sulfur‐containing solution was supplied to the surface of planar Cu foil to form CuS/Cu_2_S nanoflakes. During the following oxidation and reduction processes, the CuS/Cu_2_S layer is first oxidized to porous Cu oxide and then reduced to form a composite structure of a porous Cu layer with a dense Cu substrate. In another work, a simple slurry coating containing Cu particles, carbon nanotubes, NaCl granules, and polymer powder is selected to construct the porous conductive layer on planar Cu foil (Figure 5c).^[^ ^50^ ^]^ A porous lithiophilic and conductive porous metal current collector was obtained for the high‐performance Li‐metal anode.

#### 4.1.2 Freestanding Structure

In addition to high porosity, good electrical conductivity, and high mechanical properties, the ideal current collectors have to be self‐supporting or freestanding structures with macroscale size. Notably, the use of metal foams such as Ni, Cu, and Mo as the Li‐metal anode host could provide a high surface area and in turn to increase the Li metal loading. Typical reticulated or cellular metal foams are produced using a polyurethane foam template method which is widely applied in commercial manufacture. However, the pore size and volume are determined by the scale of polyurethane foam which usually has a low ratio of the surface area. The excessive pore size and volume can increase the Li metal loading, but they are not conducive to the efficient reversible deposition/dissolution behavior of Li metal. Hence, significant improvements have been achieved by designing micro‐/nanoporous hosts for Li‐metal anode current collectors. The assembly of metal micro‐/nanostructures is usually an efficient way to obtain PMCCs with freestanding structures. Yu and co‐workers first reported a freestanding porous metal current collector using Cu nanowire as the building block of the Li‐metal anode host (Figure 5d).^[^ ^61^ ^]^ By utilizing Cu nanowires with a large aspect ratio, PMCCs can provide an interwoven conductive network, which ensures fast electron transfer, provides homogeneous Li‐ion flux, and improves the rate performance of Li metal composite anode. In addition, the flexible Cu nanowires can adapt to the volume changes generated during Li metal deposition, contributing to uniform Li metal deposition/dissolution and effectively inhibiting the growth of Li dendrites. Besides the assembly of metal nanowires, another method to fabricate freestanding PMCCs is powder sintering. Typically, a removable template such as NaCl is mixed with copper powder followed by sintering to obtain a porous copper current collector with high porosity (Figure 5e).^[^ ^46^ ^]^ On one hand, NaCl with a high melting point does not affect the sintering reaction of Cu particles and can be easily removed, which ensures the high electrical conductivity of PMCC. On the other hand, the porous Cu with increased pore volume after the removal of NaCl significantly improves the Li metal loading. In recent years, 3D‐printed porous metal electrodes with ordered pore structures have received attention due to their wide range of adjustability and promising potential in high‐energy‐density Li‐metal anodes. As shown in Figure 5f, the Cu powder, polyvinylidene fluoride (PVDF), and 1‐methyl‐2‐pyrrolidinone are mixed to prepare a printing paste for the first step.^[^ ^65^ ^]^ For the second step, the printing paste was printed to obtain a porous Cu current collector using a homemade 3D printer. As a result, the 3D‐printed Cu structure efficiently suppresses the dendrite growth, alleviates the volume change, and exhibits long‐term cycling performance.

Overall, designing a porous metal structure with high porosity, large surface area, and high conductivity, high‐performance Li‐metal anodes could construct on these PMCCs with highly effective suppression of Li dendrites and electrochemical reversibility of Li plating/stripping. For nonfreestanding structured PMCCs, they usually show the advantages of facile preparation and low cost. However, it is well‐known that these electrodes are usually accompanied by a dense metal layer which is not conducive to and reduces the quality of the composite anode, and the porous layer on its surface is easily to fall off and is easily to be pulverized when dealing with extreme processing conditions. For freestanding structured PMCCs, their distinctive self‐supporting structure enables a promising future in the practical application of Li metal batteries. However, further development of freestanding PMCCs should consider the manufacturing cost, scalability, porosity, and processability for extreme manufacturing environments.

### 4.2 Pore Structure Design

Porous metal current collectors with various pore sizes, pore volumes, and pore distributions in their internal space greatly influence the electrochemical performance of Li‐metal anodes. It is noted that the PMCCs with neither too small nor big pore structures are appropriate for enhancing the electrochemical performance of Li‐metal anodes. The large pore can accommodate large Li metal loading and buffer the huge Li volume change but provide a limited surface area, which is not suitable for the suppression of Li dendrites. For example, among the 3D Cu current collectors, Cu foam with an ultrahigh porosity shows good potential to accommodate considerable Li metal loading. However, Cu foam as the current collector is difficult to meet the requirements for regulating the Li metal deposition behavior, such as inducing uniform deposition and suppressing the growth of Li dendrites. Moreover, the Cu foam current collector with a large pore size cannot endure the deformation of the electrode under external pressure. Therefore, the poor elasticity of Cu foam is far from enough to maintain its structural stability during deep charging and discharging. In contrast to the small pore, it can effectively decrease the local current density due to its high surface area but suffer from the poor diffusion of Li ion and volume change of Li metal. Therefore, a rational pore structure design can provide uniform Li‐ion flux, uniformly disperse the local current density of the electrode surface, release the stress generated during Li deposition, and buffer the volume expansion of Li metal, thus achieving uniform Li deposition. In this section, we carefully discuss the design of electrode structures with microscale size for PMCCs. These will be classified as disordered pore structure, ordered pore structure, and some other special pore structure designs including elastic pore, gradient pore, and bidirectional pore design.

#### 4.2.1 Disordered Pore Structure

**Figure 6:**
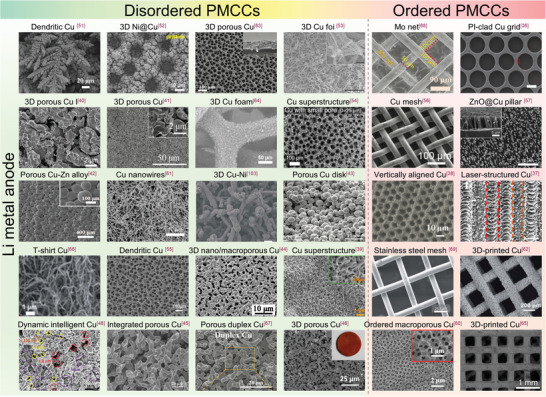
Scanning electron microscopy (SEM) images of disordered and ordered PMCCs for Li‐metal anodes. Reproduced under the terms of the CC‐BY Creative Commons Attribution 4.0 International license (https://creativecommons.org/licenses/by/4.0).^[^ ^36^ ^]^ Copyright 2018, The Authors, Published by Springer Nature. Reproduced with permission.^[^ ^37^ ^]^ Copyright 2021, American Chemical Society. Reproduced with permission.^[^ ^38^ ^]^ Copyright 2017, Wiley‐VCH. Reproduced with permission.^[^ ^39^ ^]^ Copyright 2018, Elsevier. Reproduced with permission.^[^ ^40^ ^]^ Copyright 2016, Wiley‐VCH. Reproduced with permission.^[^ ^41^ ^]^ Copyright 2018, Wiley‐VCH. Reproduced with permission.^[^ ^42^ ^]^ Copyright 2019, Elsevier. Reproduced with permission.^[^ ^43^ ^]^ Copyright 2020, Wiley‐VCH. Reproduced with permission.^[^ ^44^ ^]^ Copyright 2018, Elsevier. Reproduced with permission.^[^ ^45^ ^]^ Copyright 2022, Wiley‐VCH. Reproduced with permission.^[^ ^46^ ^]^ Copyright 2018, American Chemical Society. Reproduced with permission.^[^ ^48^ ^]^ Copyright 2020, American Chemical Society. Reproduced with permission.^[^ ^51^ ^]^ Copyright 2020, Elsevier. Reproduced with permission.^[^ ^52^ ^]^ Copyright 2018, Elsevier. Reproduced under the terms of the CC‐BY Creative Commons Attribution 4.0 International license (https://creativecommons.org/licenses/by/4.0).^[^ ^53^ ^]^ Copyright 2015, The Authors, Published by Springer Nature. Reproduced under the terms of the CC‐BY Creative Commons Attribution 4.0 International license (https://creativecommons.org/licenses/by/4.0).^[^ ^54^ ^]^ Copyright 2019, The authors, Published by Elsevier. Reproduced with permission.^[^ ^55^ ^]^ Copyright 2019, IOP Science Publishing. Reproduced with permission.^[^ ^56^ ^]^ Copyright 2017, Wiley‐VCH. Reproduced with permission.^[^ ^57^ ^]^ Copyright 2018, Wiley‐VCH. Reproduced with permission.^[^ ^60^ ^]^ Copyright 2018, Elsevier. Reproduced with permission.^[^ ^61^ ^]^ Copyright 2016, American Chemical Society. Reproduced with permission.^[^ ^62^ ^]^ Copyright 2020, The Royal Society of Chemistry. Reproduced with permission.^[^ ^63^ ^]^ Copyright 2019, Wiley‐VCH. Reproduced with permission.^[^ ^64^ ^]^ Copyright 2018, Elsevier. Reproduced with permission.^[^ ^65^ ^]^ Copyright 2021, American Chemical Society. Reproduced with permission.^[^ ^66^ ^]^ Copyright 2019, The Royal Society of Chemistry. Reproduced with permission.^[^ ^67^ ^]^ Copyright 2020, Wiley‐VCH. Reproduced with permission.^[^ ^68^ ^]^ Copyright 2019, Wiley‐VCH. Reproduced with permission.^[^ ^69^ ^]^ Copyright 2018, The Royal Society of Chemistry. Reproduced with permission.^[^ ^103^ ^]^ Copyright 2017, Elsevier.

The PMCCs with disordered pore structures can be obtained through various strategies, including chemical/electrochemical etching, high‐temperature distillation, high‐temperature sintering, slurry coating, and oxidation–reduction methods. Although the pore structure of PMCCs obtained by these widely used methods is difficult to control, the as‐prepared PMCCs usually show good processability for large‐scale production. In addition, these PMCCs with high electrochemical stability can yield a large surface area and suppress Li dendrite growth. For example, Yang and co‐workers developed a series of chemical/electrochemical etching methods to fabricate PMCCs as Li metal hosts,^[^ ^40^ ^]^ the chemical etched PMCCs acquired a high Coulombic efficiency of 97% for 250 cycles at 0.5 mA cm^−2^ in half‐cell, the Li@3D Cu composite achieved high stability in a symmetric cell for 1000 h and showed high electrochemical performance in Li@3D Cu|LiFePO_4_ full cell. The electrochemical etched PMCCs have a more accurate ability than the chemical etching method to regulate the pore structure of current collectors. As a result, the as‐prepared PMCCs with a uniform, proper pore size and smooth pore surface enable the formation of a homogeneous SEI layer, thus contributing to high electrochemical performance in half‐cells, symmetric cells, and full cells. Since the first study of the 3D porous Cu current collector was reported by Guo et al.,^[^ ^53^ ^]^ numerous studies of PMCCs have been proposed to illustrate the relationship between the performances of Li‐metal anodes and the porous structures (e.g., pore volume, pore size, and geometric shape). It is well accepted that PMCCs with a continuous conductive skeleton could facilitate the electron transfer speed. A porous Cu current collector with a duplex porous/dense configuration exhibits high performance in stabilizing Li‐metal anodes. Using vulcanization/oxidation/reduction‐assisted strategies, the porous Cu current collector was obtained with a highly continuous network and a dense substrate, enabling a highly reversible plating/stripping behavior. As a result, the duplex Cu current collector stably runs over 800 and 300 cycles at the current density of 0.5 and 1 mA cm^−2^, respectively. The assembled Li composite anode could achieve high Coulombic efficiency of 97.3% for 300 cycles and long‐term cycling life of over 880 h at 1 mA cm^−2^ with a cycling capacity of 1 mAh cm^−2^.^[^ ^67^ ^]^ Other irregular PMCCs for Li‐metal anodes are shown and summarized in **Figure** **6** and **Table** **1**.

**Table 1:** Parameters of various PMCCs for Li‐metal anodes

Current collectors		Methods for anodes	Maximum loading [mAh cm^−2^]	Half‐cell performance (current density [mA cm^−2^], areal capacity [mAh cm^−2^], cycle number [h])	Symmetry‐cell performance (current density [mA cm^−2^], areal capacity [mAh cm^−2^], life span [h])	Full‐cell performance (cathode, rate performance, and life span)	Refs.
Disordered pore structure	Dendritic Cu	Electrochemical plating	2	–	0.2, 1, 500	LiFePO_4_, 0.3 C, 40	[51]
	3D porous Ni	Electrochemical plating	3	1, 0.5, 300	–	–	[52]
	3D porous Cu	Hydrogen bubble dynamic template	2	05, 1, 250	0.2, 0.5, 600	LiFePO_4_, 2 C, 200	[63]
	3D Cu foil	Electrochemical plating	2	0.5, 1, 50	1, 1, 400	LiFePO_4_, 0.2 C, 5	[53]
	3D porous Cu	Chemical etching	2	0.5, 1, 250	0.2, 1, 1000	LiFePO_4_, 0.5 C, 300	[40]
	3D porous Cu	Electrochemical etching	2	1, 1, 200	1, 1, 400	LiFePO_4_, 1 C, 400	[41]
	3D Cu nanowire	Oxidation–reduction	3	–	10, 1, 200	LiFePO_4_, 2 C, 400	[64]
	Cu dendritic superstructure	Electrochemical plating	1	0.5, 1, 140	0.5, 1, >600	–	[54]
	3D Cu–Zn alloy	Powder sintering	5	1, 1, 160	–	–	[42]
	Cu nanowire network	Assembly	9.06	1, 1, 50	1, 1, >500	LiCoO_2_, 5 C, 100	[65]
	3D porous Ni	Powder sintering	15	2, 1, 80	1, 1, >600	LiCoO_2_, 0.2–10 C, 70	[103]
	Porous Cu disk	Powder metallurgy	9.5	–	1, 1, 1600	Lithium nickel manganese cobalt oxide (NCM), 0.5 C, 300	[43]
	TsCu	Cotton T‐shirt template	4	1, 2, 140	0.5–3, 1, >133	LiCoO_2_, 0.5–5 C, 50	[66]
	Dendritic Cu	Electrochemical plating	6	1, 1, 600	1, 1, 2400	LiFePO_4_, 1 C, 250	[55]
	3D nano‐/macroporous Cu	Powder sintering	3	1, 1, 200	0.5, 0.5, 400	LiFePO_4_, 0.5 C, 50	[44]
	3D porous Cu	Vacuum distillation	4	0.52, 1.04, 120	0.52, 0.26, 800	Li(NiCoMn)O_2_, 50 mA g^−1^, 300	[39]
	Dynamic intelligent Cu	Slurry coating	10	1, 1, 800	1, 1, 2000	LiFePO_4_, 1 C, 500	[48]
	Integrated bidirectional porous Cu	Powder sintering	7.77	1, 1, 1000	1, 1, 4000	LiFePO_4_, 1 C, 300	[45]
	Duplex porous Cu	Surface sulfur‐assisted	10	0.5, 0.5, 800	0.5, 1, 960	LiFePO_4_, 1 C, 200	[7]
	3D porous Cu	Powder sintering	7	0.5, 1, >600	1, 1, 500	LiFePO_4_, 1 C, 200	[46]
Ordered pore structure	Mo nets	Commercial product	30	–	1, 1, >833	S, 1 C, 800	[68]
	PI–clad Cu grid	Laser ablation	4.1	0.5, 0.5, 150	0.2, 0.5, 500	LiFePO_4_, 1 C, 250	[36]
	Cu mesh	Commercial product	–	0.5, 1, 100	0.5, 1, >1200	Li_4_Ti_5_O_12_, 4 C, 500	[56]
	ZnO@Cu pillar	Electrochemical plating, atomic layer deposition	2	1, 2, 100	1, 1, >100	–	[57]
	Vertically aligned Cu microchannels	Laser drilling	–	1, 3, >200	1, 1, >200	LiFePO_4_, 0.5 C, 100	[38]
	Laser‐structure Cu foil	Laser scribing	3	0.5, 0.5, 200	–	LiFePO_4_, 1 C, 600	[37]
	Stainless steel mesh	Commercial product	–	1, 1, 100	5, 5, 250	LiFePO_4_, 5 C, 500	[69]
	3D printed porous Cu	3D printing	50	–	1, 1, 500	LiFePO_4_, 1 C, 200	[61]
	Ordered microporous Cu	Inverse opal template	0.5	0.2, 0.5, 120	0.2, 0.5, >700	–	[60]
	3D printed porous Cu	3D printing	20	1, 5, >450	1, 2.5, 450	–	[62]
Special pore structure	3D Cu@PDMS	Electroless plating	5	1, 1, 200	0.25, –, >208	LiFePO_4_, 1 mA cm^−2^, 100	[70]
	3D flexible Ag nanowire	Self‐assembly	3	1, 1, 180	1, 1, 1000	LiFePO_4_, 1 C, 400	[71]
	Dynamic intelligent Cu	Slurry coating	10	1, 1, 800	1, 1, 2000	LiFePO_4_, 1 C, 500	[48]
	Integrated bidirectional porous Cu	Powder sintering	7.77	1, 1, 1000	1, 1, 4000	LiFePO_4_, 1 C, 300	[45]
	3D porous Cu	Powder sintering	5.08	1, 1, 350	0.2, 3, 800	LiFePO_4_, 1 C, 200	[47]
Lithiophilicity‐designed PMCCs	3D porous Au/Cu	Powder sintering, thermal evaporation	10	2, 1, 250	1, 1, 1300	LiFePO_4_, 1 C, 200	[72]
	3D porous CuZn alloy	Dealloying	3	0.5, 1, 220	0.5, 1, 450	–	[73]
	Zn@Cu foam	Electrochemical plating	10	1, 1, 250	1, 1, 400	LiFePO_4_, 1 C, 250	[58]
	Ag@Cu foam	Electrochemical plating	–	2, 1, >350	–	–	[59]
	3D porous CuZn alloy	Dealloying	–	0.5, 0.5, >800	0.5,1, >500	NCM811, 1 C, 500	[74]
	Eutectic gallium–indium alloy	Drop casting	50	1, 1, 400	–	S, 1 C, 60	[75]
	Ni@Li_2_O coaxial nanowire	Oxidation, electrochemical activation	4	0.5, 2, >175	0.5, 2, 1500	LiFePO_4_, 1 C, 300	[76]
	ZnO@Cu pillar	Electrochemical plating, atomic layer deposition	2	1, 2, 100	1, 1, >100	–	[58]
	Cu–CuO–Ni	Thermal oxidizing	5	1, 1, ≈250	1, 1, 580	–	[77]
	Cu_2_O@Cu foam	Plasma oxidation	10	1, 1, 250	1, 1, 3000	–	[78]
	CuO@Cu mesh	Oxidation	24	–	1, 1, 1800	NCM, 1 C, 100	[79]
	N‐doped graphene@3D Cu	Electrochemical plating	4	0.5, 4, 100	1, 2, 800	LiFePO_4_, 0.5–2 C, 100	[80]
	g‐C_3_N_4_@Ni foam	Thermal polymerization	9	2, 1, 300	1, 1, 900	LiCoO_2_, 1 C, 200	[81]
Gradient‐designed PMCC	Phosphidized Cu/Cu nanowire	Wet chemical synthesis, phosphidation	3	1, 1, 150	1, 1, 1000	LiFePO_4_, 0.5 C, 300	[83]
	3D‐Cu@Ag|Al_2_O_3_	Galvanic displacement, brush coating	2	1, 1, –	1, 1, 250	LiFePO_4_, 2 mA cm^−2^, –	[84]
	Conductive–dielectric gradient framework	Magnetron sputtering	8	0.5, 1, 600	1, 1, >750	LiFePO_4_, 1 C, 400	
	Conductivity gradient Cu nanowire	Vacuum‐assisted infiltration process	7	5, 1, 120	5, 1, 100	NCM811, 100, –	[85]
	Porous Al_2_O_3_/Ni/Au	Powder sintering, electrochemical plating, etching, thermal evaporation, magnetron sputtering	40	1, 1, 500	2, 3.5, 500	–	[12]
	Interfacial activity gradient Cu	Powder sintering	5	1, 1, 200	1, 1, >240	LiFePO_4_, rate test, ≈22	[47]

#### 4.2.2 Ordered Pore Structure

Comparing the disordered pore structure, the ordered pore structure of PMCCs is a new type of pore structure different from the disordered pore structure of PMCCs. It has an explicit structure that may take advantage of the theoretical calculations to reveal the structure–performance relationship well and offer guidance for further pore design due to the regular structure. Since the progress about using laser and 3D printing techniques to fabricate PMCCs with ordered pore structure has been introduced in Section 2 in detail, we will only discuss several different cases in this section with the highlight on the ordered PMCCs. For instance, Tang et al. developed a 3D ordered macroporous Cu (3DOM Cu) current collector using an inverse‐opal‐template method. First, monodisperse PMMA microspheres form porous templates through the self‐assembly process, then Cu was deposited on the PMMA template substrate by a chronopotentiometry method. After immersing the electrodeposition sample into acetone for 48 h to remove the PMMA microspheres, the ordered PMCCs were obtained. The 3DOM Cu current collector can stably run for 750 h at a current density of 0.2 mA cm^−2^ with a low electrochemical overpotential of <30 mV.^[^ ^60^ ^]^ Compared with the complex template method, directly using porous metal mesh to construct PMCCs with grid pore structure facilitates large‐scale preparation. For instance, Lu and co‐workers demonstrated a one‐pot facile method by using microscale Cu mesh to stabilize Li‐metal anodes. After mechanical pressing the Cu mesh into Li metal, the Li/3D Cu exhibits many advantages.^[^ ^56^ ^]^ i) The porous mesh with a suitable surface area favors the charge transfer kinetics and reduces the local current density. ii) The high conductivity of well‐connected Cu reduces the interfacial resistance and improves the rate performance. iii) The inactive Cu with high structure stability can reduce the stress induced by electrochemical deposition/dissolution and interface fluctuation, alleviating the volume fluctuation of Li. As a result, the Li/3D Cu composite anode shows a high Coulombic efficiency of 93.9% and a low voltage hysteresis of 60 mV at 0.5 mA cm^−2^, 1 mAh cm^−2^. The Li/3D Cu|Li_4_Ti_5_O_12_ full cell also shows excellent rate performance and long‐term cycling performance. All the ordered PMCCs for Li‐metal anodes introduced in this section are summarized in Figure 6 and listed in Table 1.

#### 4.2.3 Special Pore Structure

**Figure 7:**
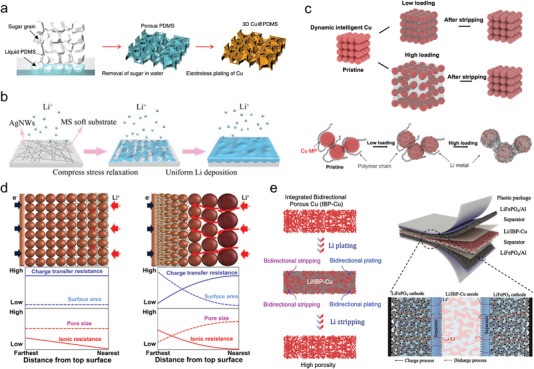
Advanced special pore structural PMCCs. a) Schematic illustration of fabrication procedures of 3D Cu/polydimethylsiloxane (PDMS) current collector. Reproduced with permission.^[^ ^70^ ^]^ Copyright 2018, Springer Nature. b) Schematic illustration of stress releasing on elastic melamine sponge@Ag NW current collector during the Li plating. Reproduced with permission.^[^ ^71^ ^]^ Copyright 2020, American Chemical Society. c) Schematic illustration of stress releasing on elastic DICu current collector and the interaction among the Cu microparticles before and after Li plating. Reproduced with permission.^[^ ^48^ ^]^ Copyright 2020, American Chemical Society. d) Schematic illustration of Li plating behavior in the normal pore and pore‐size gradient current collector. Reproduced with permission.^[^ ^47^ ^]^ Copyright 2021, American Chemical Society. e) Schematic illustration of Li plating/stripping behavior on the IBP‐Cu current collectors during Li plating and stripping. Reproduced with permission.^[^ ^45^ ^]^ Copyright 2021, Wiley‐VCH.

Except for the above‐reviewed common disordered and ordered pore structure design strategies, some other special pore architectures including elastic pore, gradient pore, and bidirectional pore have been proposed for the Li metal batteries as well. The elastic pore was first constructed for the stress relaxation of the Li‐metal anodes during the Li deposition process.^[^ ^48^ ^]^ In 2018, Jiang and co‐workers reported a 3D porous Cu current collector with elastic pores that could effectively suppress the Li dendrite growth as shown in **Figure** **7**a. It was also found that the constructed elastic pore structure could release the stress caused by Li deposition and thus affect the electrochemical performance of the anode.^[^ ^70^ ^]^ After that, strategies of the structure design toward stress relief in the Li plating/stripping process have attracted extensive attention. To study the role of Li plating residual stress in dendrite formation, they subsequently designed an elastic Cu current collector supported by a soft substrate to observe the evolution of Li during Li plating/stripping. A stress‐driven dendrite growth model was constructed to explain the residual stress relief mechanism. The 3D elastic Cu current collector design provides a new strategy to mitigate Li dendrite growth by releasing residual stress during long‐term Li plating/stripping. And the as‐obtained composite Li‐metal anodes can achieve high Coulombic efficiency of >98% for 200 cycles under a current density of 1 mA cm^−2^ and exhibit ultrahigh long‐term stability in full cells. Huang and co‐workers developed a 3D elastic current collector by compositing highly conductive silver nanowires with a lightweight melamine sponge to study the stress‐releasing mechanism of the current collector during Li plating. The silver nanowire loaded on the melamine sponge through a facile self‐assembly method exhibited high conductivity and flexibility, as shown in Figure 7b. The synergistic effect of high lithiophilicity/conductivity of silver nanowires with lightweight melamine sponges facilitates the excellent electrochemical performance of Li‐metal anodes. As a result, the elastic melamine sponge@silver nanowire (MS@Ag NW) current collector delivers a high Coulombic efficiency of 99.1% without dendrite growth. The Li–MS@Ag NW composite anodes exhibit high electrochemical stability over 1200 h with excellent structural stability and low overpotential. The full cell with Li–MS@Ag NWs shows a high discharge capacity of 119 mAh g^−1^ at a high rate of 10 C.^[^ ^71^ ^]^ In addition to introducing elastic substrates or frameworks to fabricate elastic PMCCs, employing an intelligent self‐adaptive skeleton that can dynamically alleviate the volume change of Li is another route to accommodate homogeneous Li plating/stripping (Figure 7c). For instance, by changing the granular pile structure from a compact assembled state to a relaxed stacking state, the dynamic intelligent Cu (DICu) can accommodate a high Li deposition of 10 mAh cm^−2^. Specifically, the DICu realizes a high Coulombic efficiency of 99.6% at a current density of 1 mA cm^−2^ with a lithiation amount of 1 mAh cm^−2^ for 800 cycles. Due to the high flexibility and elasticity of DICu films with high Li accommodation, residual stress induced by Li plating/stripping could be effectively alleviated, ensuring the high cyclability in symmetric and Li metal full cells.^[^ ^48^ ^]^

In addition to the elastic pore structure, regulating the gradient of the pore structure can avoid Li deposition on the top of the porous metal scaffold and improve the space utilization efficiency of PMCCs. Lee et al. compared the pore‐size gradient to study the relationship between the pore size and Li metal deposition behavior (Figure 7d).^[^ ^47^ ^]^ Specifically, the different sizes of Cu particles were selected as the building block of the pore‐size gradient PMCCs. In their pore‐size gradient PMCCs, the size of the Cu particles decreased along with the depth direction, leading to a gradually reduced pore size and increased surface area. The charge transfer resistance is differentiated along with the depth direction of the PMCCs due to the pore size and surface area differences. Thanks to the pore structure gradient, the PMCCs realize a superconformal deposition of Li metal and exhibit excellent electrochemical performance, such as stable cycling for >760 cycles at a current density of 2 mA cm^−2^ with an areal capacity of 0.5 mAh cm^−2^.

The double‐sided coated electrodes are widely used for commercial Li‐ion batteries with high energy densities. Accordingly, developing an anode current collector that can accommodate the stable and homogeneous Li plating/stripping on both sides will be highly desired for practical Li metal batteries with better performance. Hence, a bidirectional porous metal current collector with a side‐to‐side through‐pore structure is of great potential in developing a high‐performance bidirectional Li‐metal anode host. Ma and co‐workers reported an integrated bidirectional porous Cu (IBP‐Cu) current collector. Using Cu microparticles as building blocks, the IBP‐Cu obtains a through‐pore structure with high porosity (Figure 7e). By adjusting the sintering temperature, the obtained IBP‐Cu electrode with tunable pore volume and size exhibits excellent processability, facilitating large‐scale electrode preparation. The continuously porous structure from side to side of the host ensures the SEI formation with high quality, homogeneous Li metal deposition, thereby effectively suppressing dendritic Li growth. As a result, the IBP‐Cu exhibits excellent deep Li plating/stripping capability up to 7 mAh cm^−2^, a high Coulombic efficiency > 99% for 1000 cycles, outstanding rate electrochemical performance, and ultrastable long‐term cycling performance. Furthermore, the Li metal pouch cell using a single Li/IBP‐Cu composite anode and double LiFePO_4_ cathodes shows a highly elevated energy density (≈187.5%) compared with the single‐electrode‐designed pouch cells.^[^ ^45^ ^]^ In fact, integrated PMCCs with through‐pore structures are all theoretically capable of bidirectional deposition/dissolution of Li metal. Hence, it is essential to construct integrated bidirectional PMCCs that could utilize in the existing porous metal fabrication techniques for practical applications by synergistically combining with pore structure modulation, nucleation sites design, and Li deposition homogenization strategies.

### 4.3 Additional Function Design

Apart from the pore structure designs that affect the electrochemical performance of Li metal cathodes, the charge transfer and ion transport in PMCCs are also important in determining the battery performance. Specifically, the charge transfer process plays a major role in the electrochemical kinetics at a low current density.^[^ ^104^ ^]^ With the applied current densities increasing, the ion diffusion process dominates the electrochemical reaction. As discussed in Section 3.1, a high Li‐ion concentration is beneficial for prolonging the threshold time of dendrite growth and facilitating homogeneous Li nucleation. However, Li ions tend to reduce at the interface of the separator/electrode surface, resulting in a “top‐growth” Li metal deposition and even Li dendrite growth. Therefore, it is critical to rationally design the PMCC structure with functional modification to regulate charge transfer and ion diffusion. In this section, we carefully discuss the functional design of PMCCs for regulating the homogeneous Li plating/stripping behavior. These will be classified as lithiophilicity design, and functional gradient design including gradient lithiophilicity, gradient electronic conductivity, and dual gradient.

#### 4.3.1 Lithiophilicity Design

**Figure 8:**
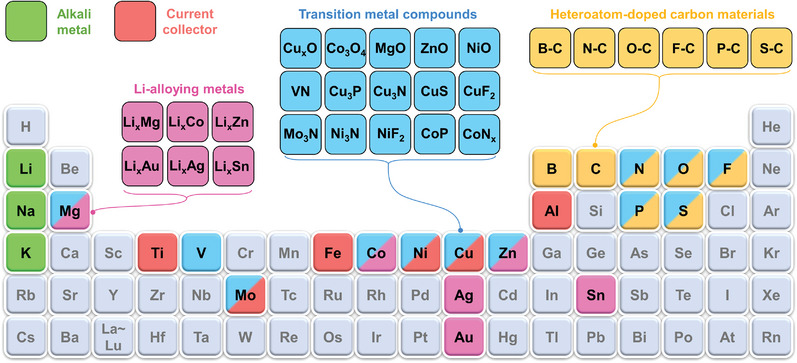
The periodic table of elements with frequently used materials for lithiophilic PMCCs.

As discussed above, the pore structure affects the ion concentration and surface charge distribution, implying that the morphology evolution of Li metal is highly dependent on the pore structure of PMCCs. However, it is not easy to differentiate the influence of the pore structure factors for the initial nucleation. To our knowledge, commonly used Cu, Ni, and stainless steel have a large Li nucleation energy barrier and do not form an alloy phase with Li because of metallic Li's unique surface energy and lattice structure. Therefore, to better understand the nucleation process of Li on the porous metal substrate, it is of great significance to introduce lithiophilic sites to induce the nucleation and uniform deposition of Li. In this section, several considerable strategies made so far on lithiophilicity design will be summarized, as shown in **Figure** **8**, including introducing Li‐alloying metals (e.g., Au, Ag, Zn, Co, Mg, Sn),^[^ ^58^, ^59^, ^72^, ^73^, ^74^, ^75^, ^105^ ^]^ using transition metal compounds that can undergo conversion reaction with Li (e.g., CuO, MgO, ZnO, NiO, VN, Cu_3_P, CuS, Mo_3_N, CoP, CoN*_x_*),^[^ ^75^, ^76^, ^77^, ^78^, ^79^, ^80^, ^81^, ^82^, ^106^ ^]^ and adopting heteroatom‐doped carbon materials (e.g., N‐doped graphene, F‐doped graphene, O‐doped graphene, F‐doped graphene, and S‐doped graphene).^[^ ^60^, ^70^, ^107^ ^]^

##### Li‐Alloying Metals

**Figure 9:**
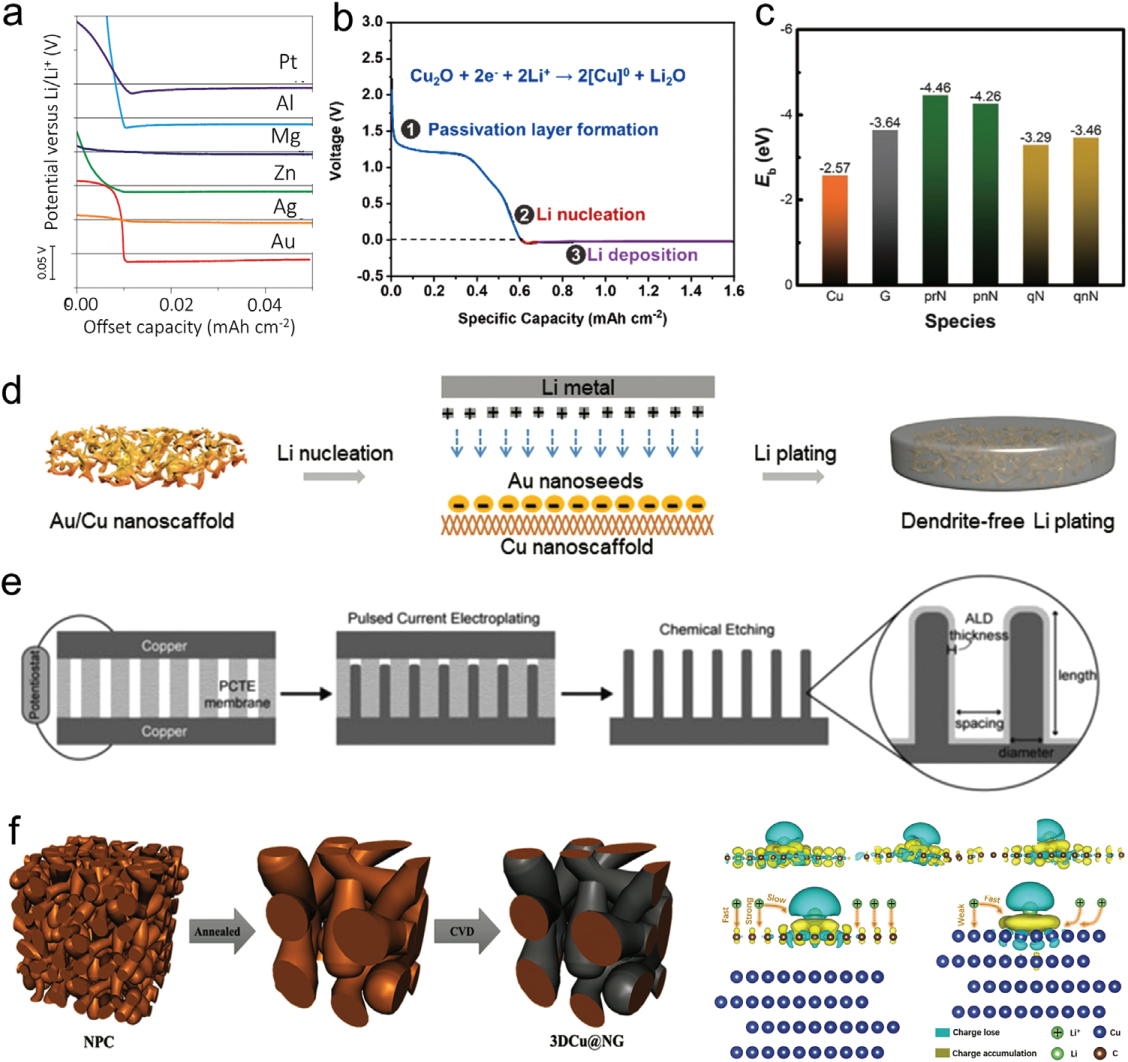
Advanced lithiophilic‐designed PMCCs. a) Nucleation overpotential curves of Li on different metal substrates. Reproduced with permission.^[^ ^105^ ^]^ Copyright 2016, Springer Nature. b) Voltage profiles of the Cu foam/Cu_2_O current collectors during the initial Li plating. Reproduced with permission.^[^ ^78^ ^]^ Copyright 2021, Elsevier. c) The binding energy of a Li atom with various materials. Reproduced with permission.^[^ ^108^ ^]^ Copyright 2017, Wiley‐VCH. d) Schematic illustration of Li plating behavior on Au/Cu current collector. Reproduced with permission.^[^ ^72^ ^]^ Copyright 2021, Wiley‐VCH. e) Schematic illustration of the fabrication of ZnO‐coated Cu current collector. Reproduced with permission.^[^ ^57^ ^]^ Copyright 2018, Wiley‐VCH. f) Schematic illustration of the fabrication of 3DCu@NG current collector, the charge density of one Li atom adsorbed on different N‐doped graphene. Reproduced with permission.^[^ ^80^ ^]^ Copyright 2018, Wiley‐VCH.

Besides adopting conventional PMCCs for stabilizing Li‐metal anodes, introducing “lithophilic” metal nanoparticles on PMCCs can reduce the nucleation obstacle of Li on its skeleton and ensure homogenous Li plating/stripping. As shown in **Figure** **9**a, Cui and co‐workers systematically studied the nucleation overpotential of Li on different metal substrates and found that no obvious nucleation happened on Au, Ag, Zn, and Mg surfaces.^[^ ^105^ ^]^ The selective deposition behavior of these lithophilic metals indicated that these metal “seeds” could induce the homogenous Li nucleation and achieve uniform deposition of Li. Based on this study, considerable efforts have been applied to incorporate lithophilic metal into PMCCs for achieving highly stable Li‐metal anodes. For instance, Lu and co‐workers developed a dealloying method to obtain a lithiophilic 3D porous CuZn current collector (Figure 9d). The residual CuZn alloys in the current collector show excellent lithiophilicity and improved homogenous Li nucleation and deposition. The as‐prepared CuZn alloy current collectors exhibited a high Coulombic efficiency of >95% at 0.5 and 1 mA cm^−2^ for 220 and 150 cycles.^[^ ^73^ ^]^ Jin and co‐workers also explored the lithophilic PMCCs with metal Au coatings for stable‐cycling Li‐metal anodes. By using a template‐sacrificed method, the atomized Cu powder fused together to form a porous interconnected Cu structure. After decorating the Au coating layer, the Au/Cu PMCCs with high porosity exhibit good lithiophilicity, facilitating even Li nucleation and deposition. Benefiting from the advantages of larger porosity and high lithiophilicity, the Li–Au/Cu composite anodes present high stability in symmetric cells with a low overpotential < 90 mV for more than 1000 h. The Li–Au/Cu|LiFePO_4_ full cells also show excellent cycling stability and long‐term cycling life.^[^ ^72^ ^]^

##### Transition Metal Compounds

Since the advent of Li‐ion batteries, the transition metal compounds (e.g., CuO, Fe_2_O_3_, Co_3_O_4_) used as the conversion reaction material for anode have been considered based on anodic redox electrochemistry.^[^ ^106^ ^]^ Compared with inactive PMCCs, the Li ions undergo conversion reactions with transition metal oxides (Figure 9b).^[^ ^78^ ^]^ Therefore, uniformly loading these metal oxides on the surface of PMCCs can induce uniform nucleation of Li. For example, Dasgupta and co‐workers reported a synergistic effect of a 3D current collector with ZnO‐modified surface layer for highly stable Li‐metal anodes (Figure 9e). After using the ALD technique to deposit a uniform ZnO layer on the surface of Cu pillar arrays, the ZnO‐coated Cu current collector exhibited no nucleation barrier without a nucleation peak during the initial Li deposition stage.^[^ ^57^ ^]^ The lithophilic reaction of ZnO could be attributed to the two‐step redox reaction, including conversion and alloying reactions

(4) $$\mathrm{ZnO}+2\mathrm{Li}↔\mathrm{Zn}+{\mathrm{Li}}_{2}O$$

(5) $$\mathrm{Zn}+x\mathrm{Li}↔{\mathrm{Li}}_{x}\mathrm{Zn}\;\left(x\leq 1\right)$$

Thanks to the synergistic effect of the ALD coating layer and appropriate pore structure, the ZnO‐coated Cu current collector shows excellent electrochemical properties involving a high Coulombic efficiency of up to 99.5%, low electrochemical overpotential, and high long‐term cycling performance. Besides, CuO nanowires can be evenly grown on the PMCCs by using a thermal‐oxidizing method. As a result, the Cu–CuO–Ni current collectors achieve a Coulombic efficiency of >95% at a current density of 1 mA cm^−2^ for more than 250 cycles and keep a stable electrochemical performance at 1 mA cm^−2^ in a symmetric cell due to the low overpotential of CuO for Li nucleation and the uniform Li^+^‐ion flux.^[^ ^77^ ^]^

##### Heteroatom‐Doped Carbon Materials

Carbon materials are widely studied as current collectors for alkali metal anodes due to their excellent physicochemical properties and abundance in microstructures and morphologies. In terms of functional groups of carbon nanomaterials, a routine method is to enhance the lithiophilicity of PMCCs by modifying heteroatom‐doped carbon materials on the surface of PMCCs (Figure 9c). Since Zhang and co‐workers reported that various types of nitrogen‐doped functional groups (i.e., pyridinic nitrogen (pnN), pyrrolic nitrogen (prN), and quaternary nitrogen (qN)) have different lithiophilicity for Li metal (Figure 9c), intensive efforts have been conducted to study N‐doped carbon materials for stabilizing Li‐metal anodes.^[^ ^108^ ^]^ For instance, Shi et al. developed a multifunctional N‐doped graphene‐modified 3D porous (3DCu@NG) current collector by a two‐step process involving chemical dealloying and chemical vapor deposition (Figure 9f). The 3DCu@NG could induce a uniform Li‐ion flux and lead to a homogenous electron distribution, finally improving the electrochemical performance. Therefore, the 3DCu@NG exhibited a low electrochemical polarization and a stable Li plating/stripping behavior. The Li–3DCu@NG composite anodes show a high Li utilization of 98% and a high Li loading of 4 mAh cm^−2^. In addition, the full cell based on the Li–3DCu@NG composite anodes and LiFePO_4_ cathode exhibited a higher rate and long‐term cycling performance than Li foil|LiFePO_4_ cells.^[^ ^80^ ^]^ Yang and co‐workers reported a graphitic‐carbon‐nitride (g‐C_3_N_4_)‐modified porous Ni foam following this motivation. The lithiophilic g‐C_3_N_4_‐modified 3D framework promoted homogenous Li deposition and suppressed the formation of Li dendrite. Combining the results of density functional theory and experimental studies, the tri‐*s*‐triazine units of g‐C_3_N_4_ quickly form a microelectric field that can generate numerous Li nucleation sites at the initial deposition stage. Benefited from the uniform Li deposition on the porous skeleton, the g‐C_3_N_4_‐modified porous Ni current collector exhibits a remarkable electrochemical performance including a high Li loading of 9 mAh cm^−2^, high Coulombic efficiency of 98% after 300 cycles with a current density of 0.5 mA cm^−2^, a low electrochemical polarization of 15 mV, and an ultralong life span for 900 h.^[^ ^85^ ^]^ After that, considerable efforts have been invested in heteroatom‐doped materials and functional groups to improve the lithiophilicity of PMCCs, including phosphorus, sulfur, oxygen, —NH_2_, —COOH, —OH.^[^ ^109^ ^]^

The strategies mentioned above mainly manipulate the initial nucleation behavior of Li metal by modifying the lithophilic metals, metal oxides, or heteroatom‐doped carbon nanomaterials on PMCCs. The introduction of a large number of nucleation sites can significantly regulate the homogeneous deposition of Li metal. However, some lithophilic metals such as Au, Ag, and Pt can effectively reduce the nucleation barrier of Li metal but their high price is not suitable for large‐scale commercial applications. Although metal oxides can induce the deposition behavior of Li metal, the capacity contributed by the conversion reaction is much smaller than the specific capacity of Li metal, and the introduction of metal oxides increases the mass of the composite anode and decreases the conductivity of the electrode. Therefore, the ideal design should introduce a lithophilic modification layer on the surface of the current collector with as low as possible mass and high conductive features to control the lithium deposition behavior and enhance the electrochemical performance of the battery.

#### 4.3.2 Functional Gradient Design

As discussed in Section 4.2.1, the lithiophilicity design not only improves the uniform Li nucleation but also increases the electroactive surface area. However, the introduction of transition metal oxide, heteroatom‐doped materials, and organic functional groups would decrease the conductivity of the porous metal substrate. To the best of our knowledge, Li prefers to deposit on top of the PMCCs due to the high conductivity, uneven Li‐ion flux, short diffusion pathway, and lithiophobicity of the metal substrate. Therefore, the rational design of PMCCs for the desired Li‐metal anode should synergistically consider the local electric field, conductivity, surface area, and lithiophilicity site. In this part, we systematically summarize recent advances in functional gradient design including lithiophilicity–lithiophobicity gradient design, conductivity gradient design, and dual gradient design for regulating Li‐ion diffusion and deposition behavior.

##### Lithiophilicity–Lithiophobicity Gradient Design

**Figure 10:**
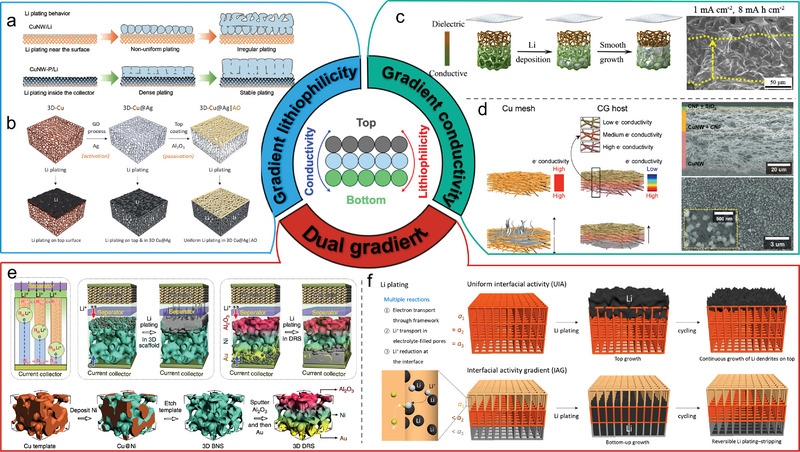
Advanced functional gradient‐designed PMCCs. a) Schematic illustration of Li plating behavior in Cu NW–P current collectors. Reproduced with permission.^[^ ^83^ ^]^ Copyright 2019, Wiley‐VCH. b) Schematic illustration of the fabrication process for 3D‐Cu@Ag|Al_2_O_3_ current collector and the Li plating behavior in 3D‐Cu@Ag|Al_2_O_3_ current collector. Reproduced with permission.^[^ ^84^ ^]^ Copyright 2019, The Royal Society of Chemistry. c) Schematic illustration of Li plating behavior in conductivity–dielectric‐gradient‐designed PMCCs, the related SEM images of the PMCC with a deposition capacity of 8 mAh cm^−2^. Reproduced with permission.^[^ ^85^ ^]^ Copyright 2018, Elsevier. d) Schematic illustration of Li plating behavior in Cu mesh and conductive‐gradient‐designed PMCCs, the corresponding cross‐sectional SEM images. Reproduced with permission.^[^ ^86^ ^]^ Copyright 2020, Wiley‐VCH. e) Schematic illustration of the bottom‐up Li plating behavior in a DRS current collector. Reproduced under the terms of the CC‐BY Creative Commons Attribution 4.0 International license (https://creativecommons.org/licenses/by/4.0).^[^ ^12^ ^]^ Copyright 2019, The Authors, Published by Springer Nature. f) Schematic illustration of the Li plating process in Cu meshes and dual gradient Cu current collector. Reproduced with permission.^[^ ^87^ ^]^ Copyright 2020, American Chemical Society.

As mentioned above, the Li metal deposition behavior is dominated by the initial nucleation. A low nucleation barrier near the surface of PMCCs is efficient for homogeneous Li metal deposition. Therefore, modifying the surface of PMCCs with lithiophilic materials, such as some metals, transition metal compounds, and heteroatom‐doped carbon materials, is an effective strategy to realize homogeneous Li nucleation and deposition. To drive the Li ion into the depth of the PMCCs, the current collector should have a suitable pore structure and a lithiophilicity–lithiophobicity gradient, namely, constructing a lithiophilic layer in the opposite direction of the Li‐ion concentration gradient. For example, Kang and co‐workers reported a Cu nanowire host with a phosphidation gradient to construct a lithiophilicity–lithiophobicity gradient for homogeneous Li metal deposition, as shown in **Figure** **10**a. The phosphating gradient balances the lithiophilicity and conductivity of the PMCCs, facilitates effective Li nucleation, enables complete Li deposition, and enhances the full utilization of the PMCCs. As a result, the PMCCs with phosphating gradient exhibit a high Coulombic efficiency of 97.3% at a current density of 1 mA cm^−2^ with a capacity of 1 mAh cm^−2^ for 150 cycles. Besides, the PMCCs with a phosphating gradient could also accommodate a high Li loading of 3 mAh cm^−2^. The corresponding specific mass capacity could achieve a high value of 1703 mAh g^−1^, much higher than graphite anodes. Moreover, long‐term cycling of 1000 and 300 h in the symmetrical cell was achieved at the current density of 1 and 2 mA cm^−2^, respectively.^[^ ^83^ ^]^ Besides, Lee and co‐workers developed a 3D‐Cu@Ag|Al_2_O_3_ framework with an activation–passivation gradient structure, enabling highly efficient and robust Li‐metal anodes (Figure 10b). The 3D porous framework provides ample space for the accommodation of Li deposition, the uniform Ag nanolayer facilitates homogeneous Li nucleation and growth, and the Al_2_O_3_ coating on the top part of the framework mitigates the top Li plating and drives the uniform Li plating in the depth of the framework. The synergistic effect of this gradient design results in high Coulombic efficiency of 98% in the half‐cell test. In addition, the assembled full cell based on 3D‐Cu@Ag|Al_2_O_3_ composite anode and LiFePO_4_ cathode exhibit reversible Li plating/stripping behavior and high rate performance.^[^ ^84^ ^]^

##### Conductivity Gradient Design

As for the conductive PMCCs, their large surface area and intrinsic high conductivity can reduce the local current density inside the interconnected pore of the electrode, thus delaying the initial threshold time for dendrite growth. Since Li nucleation always occurs where Li ions receive electrons, the Li metal tends to deposit on the top area of the electrode due to the short Li‐ion diffusion pathways from the separator to the anode and the aggregated electrical field on the top part of the electrode. Therefore, the electric field distribution also plays a significant role in guiding Li metal deposition and dissolution behavior. Constructing a conductivity–dielectric‐gradient‐designed PMCC can regulate a “bottom‐up” Li deposition and “top‐down” Li dissolution process, thus achieving high electrochemical reversibility. For instance, Yang and co‐workers developed a conductivity–dielectric‐gradient‐designed current collector by sputtering a lightweight dielectric host (melamine sponge) with a conductive Ni nanolayer, as shown in Figure 10c. Specifically, the Ni nanolayer content shows an increasing distribution from top to bottom in the melamine sponge framework due to the diffusion gradient of the Ni coating in the porous material produced by the magnetron sputtering method. This resulting conductivity–dielectric gradient electrode enabled a “bottom‐up” deposition and “top‐down” dissolution behavior. As a result, the symmetric cell exhibits a stable Li metal deposition/dissolution with a low nucleation overpotential of <20 mV for 780 h and achieves high Coulombic efficiency of 95.6% at a high current density of 8 mA cm^−2^.^[^ ^85^ ^]^ Lee and co‐workers developed an electrical conductivity gradient structure that consists of a bottom layer with a high conductive Cu nanowire network, a middle layer with an intermediate mixture, and a top layer of an electrically insulating mixture (Figure 10d). The as‐prepared conductivity gradient host enables highly reversible Li plating and stripping behaviors for more than 250 cycles at 1 mA cm^−2^ and improved electrochemical performance in symmetrical and full cells.^[^ ^86^ ^]^

##### Dual Gradient Design

In addition to considering the effects of lithiophilicity and conductivity gradient of PMCCs on Li deposition behavior separately, it is also important to synergistically combine lithiophilicity and conductivity gradients into a dual functional gradient PMCCs to further improve the Li plating and stripping behaviors by regulating the electrochemical reaction kinetics. The pioneering study of Zhang and co‐workers first demonstrated a conductivity‐ and lithiophilicity‐gradient‐designed PMCCs (deposition‐regulating scaffold, denoted as DRS) for highly stable Li‐metal anodes (Figure 10e). Specifically, by electrically passivating the top part of porous Ni to obtain a conductivity gradient and chemically activating the bottom part of porous Ni to form a lithiophilicity–lithiophobicity gradient, the Li metal realized a bottom‐up deposition behavior. The double‐function‐gradient PMCCs can not only realize a safe bottom‐up plating mode but also exhibit ultrahigh Li loading up to 40 mAh cm^−2^. Even at a high current density of 10 mA cm^−2^ and a low temperature of −15 °C, the double‐function‐gradient PMCCs still displayed stable cyclability.^[^ ^12^ ^]^ Similarly, Lee and co‐workers employed a rational dual‐function‐gradient current collector consisting of three parts: the highly conductive Cu@Ag as the lithiophilic bottom layer, bare Cu mesh as the middle layer, and the top layer containing Cu with PVDF as the passivation layer. The as‐prepared gradient current collector achieves a “bottom‐up” growth of Li metal and a much improved electrochemical performance (Figure 10f).^[^ ^87^ ^]^

Most of the reported studies on gradient‐designed PMCCs typically use the bottom‐up model for Li metal deposition in lab research. However, commercially available electrodes allow the double‐sided deposition/dissolution of Li metal. Therefore, through‐pore structural PMCCs with inside‐out gradient in Li wettability are favorable for obtaining high stability and cycling performance in practical Li metal batteries. Perhaps, future research will lead to greater opportunities, such as a triple‐gradient design of PMCCs that can synergistically modulate Li metal deposition/dissolution behavior by introducing lithiophilicity/lithiophobicity gradients, conductive/insulating gradients, and different pore‐size gradients, thus achieving optimal performance of Li metal batteries.

In conclusion, the introduction of PMCCs for Li‐metal anodes mainly provides the following functions: 1) they significantly reduce the distribution of local current density and provide uniform Li‐ion flux; 2) provide numerous protuberant tips on the conductive skeleton as the nucleation sites for guiding the uniform Li metal deposition; 3) alleviate the stress induced by Li metal deposition; 4) accommodate the significant expansion/contraction of Li metal with high porosity; 5) improve the utilization of Li metal without dendrite growth; 6) ensure the high mechanical strength, high elastic strength, and high shear modulus for commercial applications. The different host materials, fabrication methods, and battery parameters of PMCCs mentioned for Li‐metal anodes in Section 4 are listed in Table 1. Notwithstanding the present promising results, PMCCs still have great room to improve in order to meet the practical requirements, such as reducing the mass of PMCCs to improve the energy density, driving the Li ions into the depth of the PMCCs, and developing advanced fabrication strategy to improve the scalable and cost‐effective production process.

## 5 Advanced PMCCs for Na‐ and K‐Metal Anodes

Due to the natural abundance of Na and K resources, the development of Na and K metal batteries holds great potential for large‐scale application.^[^ ^27^ ^]^ Similar to the Li‐metal anode, Na‐ and K‐metal anodes also face major challenges, i.e., 1) the growth of dendrites; 2) side reactions and uncontrollable volume change during plating/stripping processes; and 3) low Coulombic efficiency and short life span. To resolve the above obstacles, PMCCs are also widely acquired in the development of Na and K metal batteries. In this section, we review the representative research progress of introducing PMCCs for Na‐ and K‐metal anodes.

### 5.1 Advanced PMCCs for Na‐Metal Anodes

**Figure 11:**
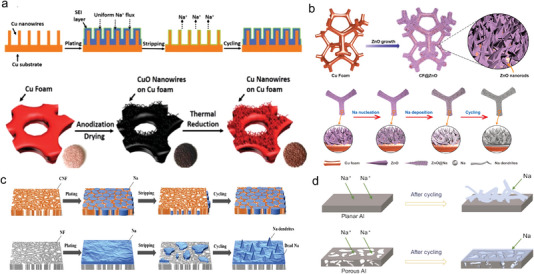
Advanced PMCCs for Na‐metal anodes. a) Schematic illustration of the Na plating behavior in Cu NW–Cu current collector, schematic illustration for the preparation process of Cu NW–Cu current collector. Reproduced with permission.^[^ ^110^ ^]^ Copyright 2018, Elsevier. b) Schematic illustration for the preparation process of CF@ZnO current collector, schematic illustration of the Na plating behavior in CF@ZnO current collector. Reproduced with permission.^[^ ^111^ ^]^ Copyright 2021, Elsevier. c) Schematic illustration of the Na plating behavior in CNF and NF current collector. Reproduced with permission.^[^ ^112^ ^]^ Copyright 2020, Wiley‐VCH. d) Schematic illustration of the Na plating behavior in porous Al and planar Al current collector. Reproduced with permission.^[^ ^97^ ^]^ Copyright 2017, American Chemical Society.

The application of PMCCs with a high surface area is conducive to reducing local current density and providing rich nucleation sites. In addition, the abundant pore structure of PMCCs promotes the sodium‐ion flux distribution, accommodates a large amount of Na deposition, and alleviates the volume change caused by repeated Na plating and stripping. Fan and co‐workers developed a Cu‐nanowire‐reinforced (Cu NW–Cu) current collector for highly stable Na‐metal anodes (**Figure** **11**a).^[^ ^110^ ^]^ By using the in situ oxidation method, the Cu nanowires grow uniformly on the surface of Cu foam. The Cu NW–Cu can provide a large surface area, afford abundant nucleation sites, accommodate ample Li storage, and reduce the local current density, leading to uniform Na plating and stripping without dendrite formation. The Na deposition capacity can be precisely controlled between 2 and 12 mAh cm^−2^. The as‐prepared Cu NW–Cu can achieve long‐term cycling beyond 1000 h and maintain high Coulombic efficiency of >97.5%. Additionally, the Cu NW–Cu delivers ultrastable cyclability over 1400 h with a low electrochemical polarization of 25 mV and a low thickness fluctuation of 2% at a current density of 1 mA cm^−2^. Applying Na/Cu NW–Cu composited anode into Na metal full batteries could deliver a high energy density of ≈442 Wh kg^−1^, enabling the practical application of high‐energy‐density Na metal batteries.

As discussed in Section 3.2, introducing lithiophilic sites is significant to homogenous Li nucleation and deposition. When it comes to the Na‐metal anodes, modulating the surface chemistry and configuration of PMCCs are also substantial for regulating the homogeneous deposition of Na metal. For example, employing ZnO nanorod arrays on a porous Cu foam (CF@ZnO) can afford abundant nucleation sites and low nucleation obstacles, facilitating the uniform nucleation and plating of Na metal (Figure 11b).^[^ ^111^ ^]^ Benefiting from these favorable merits, the as‐prepared CF@ZnO PMCCs delivered a stable Coulombic efficiency of 99% with a cycling capacity of 1 mAh cm^−2^ at a current density of 3 mA cm^−2^ for more than 300 cycles. In symmetrical cells, the Na/CF@ZnO electrode displays outstanding cycling stability with a low electrochemical polarization of 60 mV, even at a high current density of 3 mA cm^−2^. When using Na/CF@ZnO composite anode paired with a Na_3_V_2_(PO_4_)_3_ cathode in a full cell, a reversible specific capacity of 73.8 mAh g^−1^ was achieved with high Coulombic efficiency of >99.5% after 500 cycles, supporting its high potential in practical Na metal batteries.

Aside from Cu‐based PMCCs for Na‐metal anodes, nickel (Ni) metal is stable in a wide potential window range from 0 to 4 V, implying its broad applicability on both anode and cathode sides. For instance, Chen and co‐workers demonstrate a CuO‐modified Ni foam (CNF) as a stable Na metal host to guide Na metal nucleation and deposition without dendrite growth (Figure 11c).^[^ ^112^ ^]^ The CNF host is fabricated by a simple chemistry displacement reaction, showing a porous core–shell cylindrical scaffold with Cu_2_O as the shell and Ni substrate as the core. The core of CNF PMCCs offers a large pore volume, highly continuous conductive skeleton, and excellent mechanical stability, facilitating high Na deposition, high rate performance, and good processability for large‐scale fabrication, respectively. The Cu_2_O of CNF PMCCs provides abundant nucleation sites for achieving a low nucleation barrier, promoting homogeneous Na deposition. Benefiting from these advantages, the CNF PMCCs exhibit attractive electrochemical stability with a plating/stripping capacity of 1 mAh cm^−2^ at a current density of 1 mA cm^−2^ over 1000 cycles. The Na/CNF composite electrode realizes an ultrastable plating/stripping cycling performance for 2000 h with a low electrochemical polarization, much better than the pristine Na electrode. The full cell with Na/CNF composite as anode and Na_3_V_2_(PO_4_)_3_ as cathode delivers a specific capacity of 91.1 mAh g^−1^ after 300 cycles, exhibiting obviously elevated cycling performance.

The construction of Cu‐based or Ni‐based PMCCs is proved to be an effective strategy to improve the safety and stability of Na metal batteries by balancing the distribution of Na‐ion concentration and current density on the electrode surface. However, a crucial issue faced by these metal PMCCs is the high density, which will seriously restrict the improvement of the high energy density of the Na metal battery. Compared with Cu or Ni, Al metal refrains from alloying reactions with Na metal. In addition, it has a low price and lightweight characteristics, showing great potential in replacing these dense metals for high‐energy‐density Na metal batteries. Luo and co‐workers developed a porous aluminum (Al) current collector as a Na metal host (Figure 11d).^[^ ^97^ ^]^ The porous Al current collector was prepared by electrochemical corrosion of commercial Al foil to obtain a higher geometric surface area. The interconnected porous structure increases the conductivity of PMCCs and decreases the Na‐ion flux distribution, facilitating a homogeneous Na nucleation and deposition. The Na/porous Al electrode can run for over 1000 h with high Coulombic efficiency of 99.9% at a current density of 0.5 mA cm^−2^ and a cycling capacity of 0.5 mAh cm^−2^ in the symmetrical cell. The full cell using Na/porous Al as anode and O_2_ as cathode delivers a high specific capacity of ≈600 mAh g^−1^ and maintains high capacity retention after 200 cycles. The PMCCs used for Na‐metal anodes introduced in this section are summarized in **Table** **2**.

**Table 2:** Parameters of PMCCs for Na‐ and K‐metal anodes

Current collectors		Methods for anodes	Maximum loading [mAh cm^−2^]	Half‐cell performance (current density [mA cm^−2^], areal capacity [mAh cm^−2^], cycle number [h])	Symmetry‐cell performance (current density [mA cm^−2^], areal capacity [mAh cm^−2^], life span [h])	Full‐cell performance (cathode, rate performance, and life span)	Refs.
PMCC for Na‐metal anodes	CuNW–Cu	Electrochemical plating	4	1, 2, 350	1, 2, 1100	FeS_2_, 0.2 A g^−1^, 50	[110]
	Na–Sn alloy and Na_2_O framework	Melting SnO_2_ with Na	8	–	1, 1, 700	Na_3_V_2_(PO_4_)_3_, 1 C, 300	[113]
	3D porous Cu	Chemical dealloying	3	1, 1, 400	1, 1, 2000	Na_3_V_2_(PO_4_)_3_, 1 C, 100	[114]
	3D Cu nanowires/Cu foil	Hydrothermal synthesis	–	1, 3, 100	0.5, 0.5, 250	Na_3_V_2_(PO_4_)_3_, 1 C, 200	[115]
	O‐treated Cu foam	Thermal melt infusion	–	–	2, 3, 300	Na_3_V_2_(PO_4_)_3_, 5 C, 100	[116]
	Porous Al	Electrochemical plating	–	1, 0.5, 1200	0.5, 0.5, 1000	Na_3_V_2_(PO_4_)_3_, 0.1 mA cm^−2^, 350	[97]
	CuO/Cu_2_O@Cu foam	Oxidation–dehydration	7.5	–	0.5, 0.5, 1000	NaTi_2_(PO_4_)_3_, 1–100 C, 45	[117]
	ZnO@Cu foam	Chemical precipitation	10	1, 1300	1, 1350	Na_3_V_2_(PO_4_)_3_, 10 C, 500	[111]
	CuO@Ni	Thermal melt infusion	–	–	1, 1, 2000	Na_3_V_2_(PO_4_)_3_, 5 C, 100	[112]
PMCC for K‐metal anodes	rGO@3D‐Cu	Self‐assembly	–	0.5, 1, 100	0.5, 0.5, ≈163	–	[118]
	Cu_3_Pt–Cu mesh	Galvanic replacement	5	–	0.5, 2, >300	Prussian blue, 0.1 A g^−1^, 250	[119]
	Porous Al@Al	Powder sintering	2	0.5, 1, 1000	0.5, 0.5, ≈1900	–	[92]

PMCCs offer apparent advantages such as high mechanical strength, excellent electrical conductivity, and satisfactory processability. However, their high densities in the battery systems will largely reduce the energy density, limiting their practical application. In addition, highly reactive Na metal is more likely to induce side reactions and uneven deposition on the anode surface than Li metal. Therefore, reducing the densities of PMCCs while decorating their surface with sodiophilic layers and/or highly stable artificial SEI layers should be more effective to improve the electrochemical performance of Na‐metal anodes.

### 5.2 Advanced PMCCs for K‐Metal Anodes

Although the gravimetric capacity of K metal (685 mAh g^−1^) is lower than Li and Na due to its higher atomic weight in nature, it still has various advantages, such as lower density and redox potential of −2.93 V (vs standard hydrogen electrode (SHE)) than Na metal, implying that K metal batteries could achieve a high energy density and operate at a wide voltage window, respectively.^[^ ^28^ ^]^ In addition, K metal shows a tremendous competitive advantage compared with the rising Li‐ and Na‐metal anode due to its low cost and high abundance. Moreover, the melting point of K (≈63.5 °C) is much lower than Li metal (≈180 °C) and Na metal (≈98 °C), which can be easily used for fabricating K‐metal composite anode through the thermal melting infusion method. As we discussed throughout the text, the unstable SEI layer and dendrite growth are always significant issues for alkali metal anodes. In terms of K‐metal anode, the nucleation of K metal is nonplanar, i.e., isolated and island‐like, hence the local current density on the surface of the isolated region will be enhanced, resulting in the generation of dendrites seriously. Actually, the challenge for K metal is more severe as compared with Li or Na metal due to its severely reactive with electrolytes and thermodynamically instability with standard battery current collectors. In addition, the intrinsic potassiophilicity of K metal on the Cu host is poor, implying that no matter on a planar or porous Cu current collector, the K metal always exists in inhomogeneous deposition state. Advanced efforts have been done to employ PMCCs with tuned surface chemistry to bolster the electrochemical stability of K‐metal anode to address these issues.

**Figure 12:**
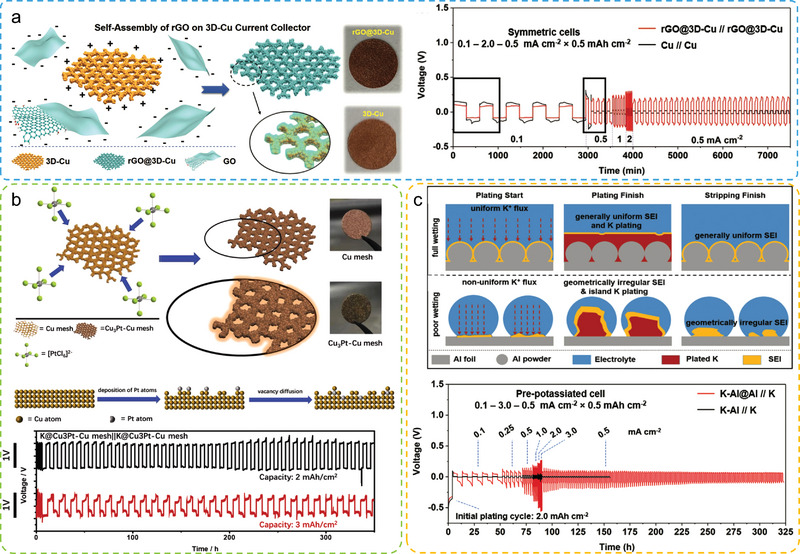
Advanced PMCCs for K‐metal anodes. a) Schematic illustration for the preparation process of rGO@3D‐Cu current collector, the electrochemical performance of K/rGO–3D‐Cu|K/rGO–3D‐Cu symmetric cell. Reproduced with permission.^[^ ^118^ ^]^ Copyright 2019, Wiley‐VCH. b) Schematic illustrations of fabrication of Cu_3_Pt–Cu mesh, the electrochemical performance of K@Cu_3_Pt–Cu mesh|K@Cu_3_Pt–Cu symmetric cell. Reproduced with permission.^[^ ^119^ ^]^ Copyright 2020, Elsevier. c) Schematic illustration of the electrolyte wetting effect on the Al@Al and planar Al current collector during plating and stripping. The electrochemical performance of K–Al@Al|K and K–Al|K symmetric cell. Reproduced with permission.^[^ ^98^ ^]^ Copyright 2020, Wiley‐VCH.

Mitlin and co‐workers developed a 3D Cu current collector with a partially reduced graphene oxide (rGO) coating layer to create a potassiophilic surface (**Figure** **12**a).^[^ ^118^ ^]^ The potassiophilic versus potassiophobic experiment was conducted by melting K metal into PMCCs. The molten K can thoroughly wet rGO@3D‐Cu current collector in seconds but does not wet the unfunctionalized porous Cu current collector, demonstrating the significant improvement of K wetting behavior (i.e., potassiophilicity). The rGO@3D‐Cu current collector exhibits low electrochemical polarization and high Coulombic efficiency in a half‐cell system, directly corresponding to a highly stable SEI and dendrite‐free K‐metal anode. The K/GO@3D‐Cu electrode also shows a stable K plating/stripping behavior at 0.1–2 mA cm^−2^ in the symmetrical cell.

In addition to the rGO mentioned above, the alloy can also create abundant nucleation sites to reduce the nucleation overpotential and induce homogeneous K metal deposition. Zhang and co‐workers developed potassiophilic Cu_3_Pt‐alloy‐modified porous Cu by a fast and straightforward galvanic replacement reaction.^[^ ^119^ ^]^ The single Pt atom was deposited on the surface of the Cu at the expense of double Cu atoms according to the reaction stoichiometry, which will produce abundant defects. The Cu atoms form an alloy phase with the deposited Pt atoms through vacancy diffusion to reduce the increased surface energy. The binding energy of the K atom with Cu and Cu_3_Pt are calculated to be −1.74 and −2.68 eV, respectively, indicating that Cu_3_Pt has a high potassiophilicity to K metal (Figure 12b). The Cu_3_Pt–Cu current collector exhibited a Coulombic efficiency of 95% for 150 cycles at a current density of 0.5 mA cm^−2^ with a cycling capacity of 1 mAh cm^−2^. The K/Cu_3_Pt–Cu electrodes deliver an electrochemical polarization of 1000 mV for more than 300 h at a cycling capacity of 2 and 3 mAh cm^−2^, indicating the excellent cycling stability of Cu_3_Pt–Cu PMCCs for high‐capacity applications.

Apart from the modified Cu‐based PMCCs, Al also could be applied in K‐metal anodes due to its nonalloying reaction with K metal at low potential. Mitlin and co‐workers developed a 3D unique all‐Al porous (Al@Al) current collector through sintering Al powder on a dense Al foil (Figure 12c).^[^ ^98^ ^]^ The Al@Al current collector increased the surface area, and improved the electrolyte wettability, thereby enhancing the transport rate of the K ion in electrolyte and inducing uniform K metal deposition without dendrite growth. The Al@Al and K/Al@Al exhibit a low and stable electrochemical polarization, indicating that the porous Al structure is beneficial for the stable K plating/stripping kinetics and the formation of a stable SEI layer. As a result, the Al@Al current collector delivers a high Coulombic efficiency of 98.9% at a current density of 0.5 mA cm^−2^ with a cycling capacity of 2 mAh cm^−2^. The K/Al@Al electrode exhibits low electrochemical overpotential and high electrochemical stability in a wide current density range, e.g., 0.178 V at 3 mA cm^−2^ for 130 cycles (2600 min).

Reversible K‐metal stripping and plating can be achieved by introducing PMCCs, making K‐metal anodes promising for practical applications. In addition, taking advantage of the low melting point of K metal, K–PMCC composite anodes could be fabricated by the low‐temperature melting method to meet mass production requirements. However, due to the ultrahigh reactivity of K metal, studies on K‐metal anodes are far behind those of Li‐ and Na‐metal anodes. Therefore, using K‐metal anodes requires synergistic developments with structural design, electrode/electrolyte interface, and electrolyte engineering for practical applications. Following the effective strategies of Li‐ and Na‐metal anodes, the high stability and high electrochemical performance of K‐metal anodes are likely to improve.

## 6 Summary and Outlook

**Figure 13:**
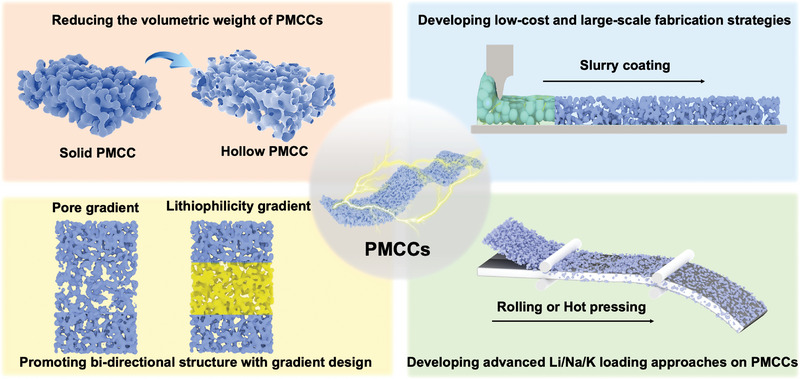
Perspectives for future studies on the design of porous metal current collectors.

Alkali metal batteries are promising energy storage devices, though some challenges remain on the way of their commercialization, like dendrite growth, unstable SEI layer, and low Columbic efficiency. PMCCs with porous structure, sufficient inner space, large surface area, tunable conductivity and pore size, and metallophilicity have been proven as effective means to alleviate PMCC challenges. This paper reviews the recent progress in developing advanced PMCCs for stabilizing alkali metal anodes. Special attentions have been paid to the advances in fabrication approaches, working mechanisms, and correlating structure–property relationships to optimize their electrochemical performance. Though significant progress on improving the performance of alkali metal anodes has been achieved, more needs to be done. Based on our understanding, the primary efforts in the future should be dedicated to the following aspects to achieve highly stable, ultralight, and economically scalable PMCCs, as shown in **Figure** **13**.

1) More fundamental research on the SEI formation in PMCCs for alkali metal anodes is needed. The formation and stability of SEI on 3D current collectors and 2D current collectors need to be studied in detail. PMCCs with a relatively large surface area will consume a large amount of electrolyte to form more SEI layers. Hence, some electrolyte additives, such as fluoroethylene carbonate and vinylene carbonate are necessary for stabilizing the SEI layer.^[^ ^120^ ^]^ Most research on PMCCs used button cells to evaluate the electrochemical battery performance. In this case, even if the SEI undergoes repeated formation and destruction, the battery performance will not be significantly affected due to the large excess amount of electrolytes. Thus, the electrochemical performance and degradation mechanism of PMCC‐based anodes at lean electrolyte usage should be tested. In addition, combining PMCCs with artificial SEI or solid/quasi‐solid electrolytes could offer reasonable solutions to improve battery performance.
2) In the currently reported studies, many PMCCs are constructed on solid metal structures, such as metal powders or derivatives. It will increase the mass of additional inactive materials in the current collector while reducing the space for loading alkali metals. In future research, PMCCs composed of the hollow skeleton should be developed to decrease the amount of inactive mass and increase the porosity of the current collector, which can effectively enhance the overall energy density of the alkali metal anode. For example, with a fixed anode thickness, the PMCCs must theoretically have a porosity of 50% or more to accommodate Li metal to compete with commercial graphite in theoretical capacity. Transforming solid‐structured PMCCs into hollow‐structured PMCCs or constructing ultralight structured porous metal aerogels may be an effective way to increase the porosity of the current collectors. However, it should be noted that increasing the porosity of the current collector will inevitably affect the mechanical properties of the current collector. Therefore, balancing the porosity and mechanical strength is essential in designing PMCCs.
3) At present, various methodologies have been adopted for PMCC preparation and alkali metal loading. Some of these techniques (e.g., laser scribing for PMCC preparation and electrodeposition for alkali metal loading) are indeed exemplary methods for mechanistic investigations aimed at a fundamental understanding of PMCC behavior. However, when considering future mass production, some of the approaches are not suitable either from the environmental or economic point of view. Therefore, developing more eco‐friendly, cost‐effective, and scalable current collector preparation methods is crucial. Among them, the slurry coating technique, one of the traditional methods of electrode preparation for Li/Na batteries, is perhaps the most suitable technique for practical large‐scale applications. Note that almost all reported PMCCs employ electrochemical deposition methods to construct composite anodes. In the future, the preparation method of the composite anode is required to comprehensively evaluate the complexity of the preparation process, cost, and feasibility of large‐scale development. The hot pressing method might be a good choice for fabricating alkali metal composite anodes.
4) Current research on PMCCs is mainly focused on using one‐side current collectors to improve the stability of alkali metal anodes. More activities should be focused on a more realistic situation, where both sides of the current collector will be loaded with active material for battery assembly. Thus, it is essential to prepare PMCCs with double‐sided coating or perforated pore engineering and test their performances on a larger scale, e.g., using pouch cell and battery modules.
5) Next‐generation batteries such as aqueous‐metal (Zn, Mg, Ca, and Al)‐based batteries are promising energy‐storage systems for future smart grids.^[^ ^121^, ^122^, ^123^, ^124^ ^]^ The PMCCs in such systems might bring more opportunities, e.g., accommodating highly active materials loading, suppressing the metal dendrites growth, and improving electrochemical performance. However, taking the aqueous Zn metal battery as an example, Zn metal shows intrinsic thermodynamic instability in the aqueous system. PMCCs with large porosity will accommodate the deposition of Zn with a large specific surface area, which might cause more serious side reactions. Hence, designing a composite anode that synergistically combines PMCCs with the functional layer of side reaction depression is worth future exploration.
6) Future research of PMCCs coupled with artificial intelligence (AI) technology, especially the structure optimization algorithms, is highly expected for designing smart PMCCs and batteries. For instance, employing AI technology and integrating the structure optimization algorithm into a battery's fabrication system can predict the well‐designed material modeling and create the best electrode structure according to the application requirements. In addition, the optimization algorithm of AI is beneficial for designing the optimal configuration of batteries, explaining the battery's reaction mechanism, and simulating the potential thermal runaway behavior, finally delivering better performance and safety in the next‐generation high‐energy‐density battery.

With the development of nanotechnology, material design, and characterization techniques, as well as a deepener understanding of relative mechanisms in alkali metal anodes, it is reasonable to believe that more extraordinary breakthroughs can be made in this field soon.

## Conflict of Interest

The authors declare no conflict of interest.
